# Evaluation of freely available software tools for untargeted quantification of ^13^C isotopic enrichment in cellular metabolome from HR-LC/MS data

**DOI:** 10.1016/j.mec.2019.e00120

**Published:** 2019-12-26

**Authors:** Manohar C. Dange, Vivek Mishra, Bratati Mukherjee, Damini Jaiswal, Murtaza S. Merchant, Charulata B. Prasannan, Pramod P. Wangikar

**Affiliations:** a Department of Chemical Engineering, Indian Institute of Technology Bombay, Powai, Mumbai, 40076, India; b DBT-Pan IIT Center for Bioenergy, Indian Institute of Technology Bombay, Powai, Mumbai, 400076, India; c Wadhwani Research Center for Bioengineering, Indian Institute of Technology Bombay, Powai, Mumbai, 400076, India

**Keywords:** ^13^C metabolic flux analysis, *Synechococcus* sp. PCC 7002, Cyanobacteria, Reticulocytes, Methanolicus, Untargeted analysis

## Abstract

^13^C Metabolic Flux Analysis (^13^C-MFA) involves the quantification of isotopic enrichment in cellular metabolites and fitting the resultant data to the metabolic network model of the organism. Coverage and resolution of the resultant flux map depends on the total number of metabolites and fragments in which ^13^C enrichment can be quantified accurately. Experimental techniques for tracking ^13^C enrichment are evolving rapidly and large volumes of data are now routinely generated through the use of Liquid Chromatography coupled with High-Resolution Mass Spectrometry (HR-LC/MS). Therefore, the current manuscript is focused on the challenges in high-throughput analyses of such large datasets. Current ^13^C-MFA studies often have to rely on the targeted quantification of a small subset of metabolites, thereby leaving a large fraction of the data unexplored. A number of public domain software tools have been reported in recent years for the untargeted quantitation of isotopic enrichment. However, the suitability of their application across diverse datasets has not been investigated. Here, we test the software tools X^13^CMS, DynaMet, geoRge, and HiResTEC with three diverse datasets. The tools provided a global, untargeted view of ^13^C enrichment in metabolites in all three datasets and a much-needed automation in data analysis. Some inconsistencies were observed in results obtained from the different tools, which could be partially ascribed to the lack of baseline separation and potential mass conflicts. After removing the false positives manually, isotopic enrichment could be quantified reliably in a large repertoire of metabolites. Of the software tools explored, geoRge and HiResTEC consistently performed well for the untargeted analysis of all datasets tested.

## Highlights

• X^13^CMS, DynaMet, geoRge and HiResTEC were evaluated for untargeted and targeted analysis.
• These software tools provided a quick global view of ^13^C enrichment in the metabolome.
• Extensive parameter optimization and manual curation of results were necessary.
• geoRge and HiResTEC worked well for untargeted analyses of dynamic labeling datasets.

## Graphical abstract

**Figure:**
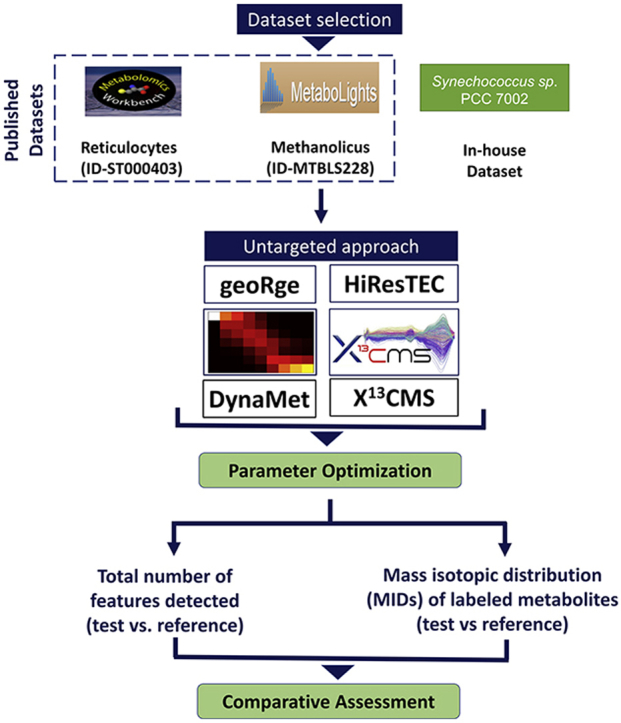

## Abbreviations

^13^C MFA: ^13^Carbon Metabolic Flux Analysis
MID: Mass Isotopologue Distribution
HR-LC/MS: Liquid Chromatography coupled to High-Resolution Mass Spectrometry
RT: Retention Time
m/z: Mass to Charge ratio
3-PGA: 3-Phosphoglyceric acid
ADP: Adenosine diphosphate
ADP-G: ADP-Glucose
ATP: Adenosine triphosphate
G3P: Glyceraldehyde-3-phosphate
G6P: Glucose-6-phosphate
PEP: Phosphoenolpyruvate
R5P: Ribulose 5-Phosphate
S7P: Sedoheptulose-7-phosphate
UDP-G: Uridine diphosphate-Glucose
RMSD: Root Mean Standard Deviation
XIC: Extracted Ion Chromatogram
MS1 & MS2: Stages of data generation in tandem mass spectrometry
GUI: Graphical User Interface

## 1 Introduction

Metabolomic studies with stable isotopic tracers have made significant contributions in furthering our understanding of cellular metabolism. Based on the study objective, these can broadly be categorized as (i) Qualitative, global metabolomics studies that aim to obtain binary data on the presence/absence of metabolites, (ii) Quantitative metabolomics with the goal to achieve absolute or relative quantitation of a large number of metabolites, (iii) Metabolic pathway analysis to discern between alternative pathways and (iv) Quantitative, ^13^C Metabolic Flux Analysis (^13^C-MFA). The requirements of accurate identification and quantification of metabolites and their isotopologues become more and more stringent as we go from the first to the fourth type of study. The knowledge gained through the study of metabolic fluxes is often critical to the comprehensive understanding of the factors governing the phenome of a biological system and for the construction of designer microbes with applications in biotechnology (Wiechert, 2001) (Zamboni and Sauer, 2009) (Young, 2014) (Jawed et al., 2019). The conventional, stationary state ^13^C-MFA technique involves feeding cultures with isotopically labeled substrate and quantification of the label incorporation in terminal metabolites such as the proteogenic amino acids at isotopic steady state (Alagesan et al., 2013). For the newer, non-stationary ^13^C MFA approach, the dynamics of incorporation of the isotopic label in intermediate metabolites and their fragments need to be monitored (Young et al., 2011) (Hendry et al., 2017) (Prasannan et al., 2019). Therefore, quantification of ^13^C enrichment in a wider array of metabolites and their fragments would facilitate the generation of high-resolution flux maps (Choi and Antoniewicz, 2011) (Choi and Antoniewicz, 2019). Until recently, non-stationary ^13^C MFA has been carried out with dynamic enrichment data for a handful of central carbon metabolites. However, there is a push towards obtaining labeling data for a larger number of intermediate metabolites (Jaiswal et al., 2018). Fluxomics or ^13^C-MFA conducted on a global metabolome in a high throughput manner, would therefore be a valuable tool to complement data available from other -omics studies (Hoffmann et al., 2018).

Liquid Chromatography coupled with high resolution Mass Spectrometry (HR-LC/MS) allows the collection of a large amount of spectral data for each sample, and has therefore emerged as a powerful tool for metabolomics studies (Spicer et al., 2017). Such data is considered to be suitable for untargeted analysis of hundreds of metabolites (Gowda et al., 2014). Several software tools such as XCMS online (TautenhahnRalf. et al., 2013) and MetaboAnalyst (Xia et al., 2009) provide automation in peak picking, comparison of metabolite intensities across samples, statistical tests and connection with other metadata, such as metabolic pathways. Much of this untargeted analysis is often limited to monoisotopic peaks. HR-LC/MS data from a typical ^13^C labeling experiment has information on the ^13^C label incorporation in a wide range of metabolites. Suitable software tools that can perform an untargeted analysis to capture these labeled metabolites have been lacking. Because of this, such data is typically analysed in a targeted manner using vendor provided licensed software packages accompanying the LC-MS instrument used. This limits the coverage of metabolites and requires prior knowledge of their chromatographic retention times (RT) and mass to charge ratio (m/z) values. However, several freely available software tools have recently been developed to provide automation in detection and quantification of isotopologues of cellular metabolites in an untargeted manner in ^13^C labeled datasets. These include X^13^CMS (Patti and CMS, 2014), geoRge (Capellades et al., 2016), DynaMet (Kiefer et al., 2015), mzmatch-ISO (Chokkathukalam et al., 2013), MetExtract II (Bueschl et al., 2017), and HiResTEC (Hoffmann et al., 2018). These tools differ in algorithms used for the key steps of analysis such as peak picking, grouping of potential isotopologues, and statistical evaluation of the data. Together, these contribute towards a software’s ability to detect the maximum number of true features while minimizing false positives. These tools have been evaluated individually with datasets generated by the developers of the respective software tools. Each of these software claim to provide specific improvements to the quality and reliability of both targeted and untargeted analyses. However, the suitability of these software for the analysis of datasets generated in different laboratories using diverse biological samples and data acquisition platforms remains largely unexplored.

All six freely available software tools were initially explored. However, due to either technical difficulties in installation (mzMatch-ISO), or the infeasibility of their use with the selected datasets (MetExtract II), some tools were left out from the study design. In this study, we have therefore evaluated the applicability of four selected software tools, *viz*., X^13^CMS, geoRge, HiResTEC and DynaMet towards the untargeted quantification of ^13^C enrichment in three different biological datasets, each comprising of a time-course ^13^C labeling experiment. We find that each of these software tools is indeed able to detect and quantify a large number of chromatographic features that accumulate the ^13^C label, including a substantial number of new features. We have attempted to provide an independent assessment of these software tools from an end user’s perspective. We believe that our results and recommendations will be useful not only to potential users but also future developers of such software tools.

## 2 Experimental section

### 2.1 Materials

All chemicals used were purchased from Sigma Aldrich (St. Louis, MO) and Merck (Burlington, MA).

### 2.2 *Growth,*^*13*^*C labeling and metabolite extraction for Synechococcus* sp *PCC 7002*

The cyanobacterial strain *Synechococcus* sp. PCC 7002 (henceforth referred to as *Synechococcus* sp.) was cultured as previously reported (Hendry et al., 2017). The samples were collected and quenched at 0, 60, 120, 180, 240, 300, 900 and 1800 ​s following the introduction of ^13^C sodium bicarbonate. However, for targeted analysis, time points up to 240s were used in order to capture the transient labeling in intermediate metabolites. The samples were quickly filtered and metabolites extracted from the cells using the method previously described (Hendry et al., 2017).

### 2.3 Data acquisition and analysis of datasets

#### 2.3.1 Synechococcus sp. dataset

This dataset was acquired using UHPLC (Nexera LC-30 AD, Shimadzu, Kyoto, Japan) coupled with a Triple TOF 5600 ​+ ​mass spectrophotometer (SCIEX, Framingham, MA). This dataset has been submitted to Metabolomics Workbench (https://doi.org/10.21228/M87384). Chromatographic separation was achieved by injecting 10 ​μL sample on a C-18 synergi-hydro RP column (Phenomenex, Torrance, CA) as described previously using an ion-pairing reagent (Lu et al., 2010). The mobile phases used were, solvent A: 10 ​mM tributylamine and 11 ​mM acetic acid and solvent B: methanol. The gradient program was as follows, t ​= ​0 ​min, 0% B; t ​= ​2 ​min, 0% B; t ​= ​8 ​min, 35% B; t ​= ​10.5 ​min, 35% B; t ​= ​15.5 ​min, 90% B; t ​= ​20.5 ​min, 90% B; t ​= ​22 ​min, 0% B, t ​= ​30 ​min, 0% B at a flow rate of 0.3 ​mL/min and column temperature 25 ​°C (Prasannan et al., 2018). The *Synechococcus* sp dataset comprised of two technical replicates (n ​= ​2). MS data was collected in negative ion mode with an ion spray voltage of 4500 ​V and interface heater temperature of 450 ​°C. Both ion source gases GS1 (gas 1) and GS2 (gas 2) were set at 40 psi while the curtain gas was set at 35 psi. Untargeted analysis was performed using geoRge (Capellades et al., 2016), DynaMet (Kiefer et al., 2015) and HiResTEC (Hoffmann et al., 2018) while a proprietary software, MultiQuant^TM^ 3.0.1 (SCIEX, Framingham, MA), was used for the targeted analysis. Peak areas for the monoisotopic peaks and the isotopologues were integrated for the respective mean m/z values and m/z tolerance of ±0.05 ​Da and retention time window of ±30 ​s. The options for Gaussian smoothing and baseline subtraction were enabled. The fractions for the isotopologues were calculated from the respective peak areas obtained at a specific time point.

Raw files for this dataset was available in .wiff and .wiff.scan formats which were then converted to .mzXML format using ProteoWizard (version 3.0.10875) (Kessner et al., 2008). The parameter settings for conversion included the default signal to noise ratio and minimum peak spacing (both set as 0.1) with the peak picking filter set for continuous wavelet transform algorithm at MS1 level. These converted .mzXML files were then used for analysis with X^13^CMS, geoRge, DynaMet, and HiResTEC.

#### 2.3.2 Bacillus methanolicus dataset

This dataset consists of ^13^C labeled data from the model strain *Bacillus methanolicus* MGA3 (henceforth referred to as Methanolicus), grown in the presence of ^13^C methanol (Kiefer et al., 2015). It included two biological replicates, (.mzML files available at the MetaboLights repository, study ID – MTBLS228). Metabolites were extracted from samples quenched at 0, 5, 10, 20, 30, 60, 120, 300, and 600s time points after the introduction of the ^13^C tracer. For this dataset X^13^CMS (Patti and CMS, 2014), geoRge (Capellades et al., 2016) and HiResTEC (Hoffmann et al., 2018) were used as the test software for comparative analysis with the reference software, DynaMet (Kiefer et al., 2015).

#### 2.3.3 Human-stem-cell-derived reticulocytes dataset

This dataset was generated to study the metabolic networks active in reticulocytes, which would eventually mature to form erythrocytes (Srivastava et al., 2017). It contains data from the LC/MS analysis of metabolite extracts of labeled erythrocytes and reticulocytes. This dataset is available on the Metabolomics Workbench repository (Project ID – PR000315, Study ID – ST000403). The details of the chromatography method and MS analysis have been described in detail (Srivastava et al., 2017). Raw files in the repository include replicate data (n ​= ​3) for samples labeled with 50% uniformly labeled ^13^C glucose. Two time points were considered (1 and 20 ​h), and data was acquired in polarity switching mode (alternative positive ion and negative ion mode-based acquisition). The data for reticulocytes was downloaded and converted to .mzXML format using ProteoWizard (version 3.0.10875) (Kessner et al., 2008). As these files contained information from both positive and negative ion modes, we processed the .mzXML conversion by setting a negative polarity filter through the command line. This allowed segregation of the negative ion data in converted files. This was necessary as a direct conversion without setting the polarity filter affected analysis. Apart from the raw data files, a complete list of features detected in untargeted analysis using mzMatch-ISO (Chokkathukalam et al., 2013) (reference software for this dataset) is available together with the mass isotopic distribution for a few metabolites provided as supplementary information (Srivastava et al., 2017). This information helped to create a benchmarked list for comparison, when the dataset was processed with the test software, X^13^CMS (Patti and CMS, 2014), geoRge (Capellades et al., 2016) and HiResTEC (Hoffmann et al., 2018).

### 2.4 Installation and usage of software packages

The software tools X^13^CMS (Patti and CMS, 2014), geoRge (Capellades et al., 2016), DynaMet (Kiefer et al., 2015), and HiResTEC (Hoffmann et al., 2018) were installed using guidelines provided by their respective developers. R based packages (X^13^CMS, geoRge, and HiResTEC) required the prior installation of the XCMS package (Smith et al., 2006) for pre-processing of data. DynaMet was the only software that provided a graphical user interface for the input of parameters while others required software specific scripts. For each software tool, parameters used by their respective developers were used as a default for the initial untargeted analysis. Parameters were subsequently optimized to capture the maximum number of features possible, through a manual inspection of the feature list for the presence of targeted metabolites and their isotopologues. Mass Isotopologue Distribution (MID) profiles were generated manually for each feature after exporting data regarding the intensities/peak areas of its isotopologues onto an xlsx spreadsheet. For the visual estimation of peak quality, Extracted Ion Chromatogram (XIC) of each feature was generated using XCMS with a tolerance of 0.05 ​Da and a retention time range of 4 ​min.

## 3 Results and discussion

### 3.1 Study design

**Fig. 1:**
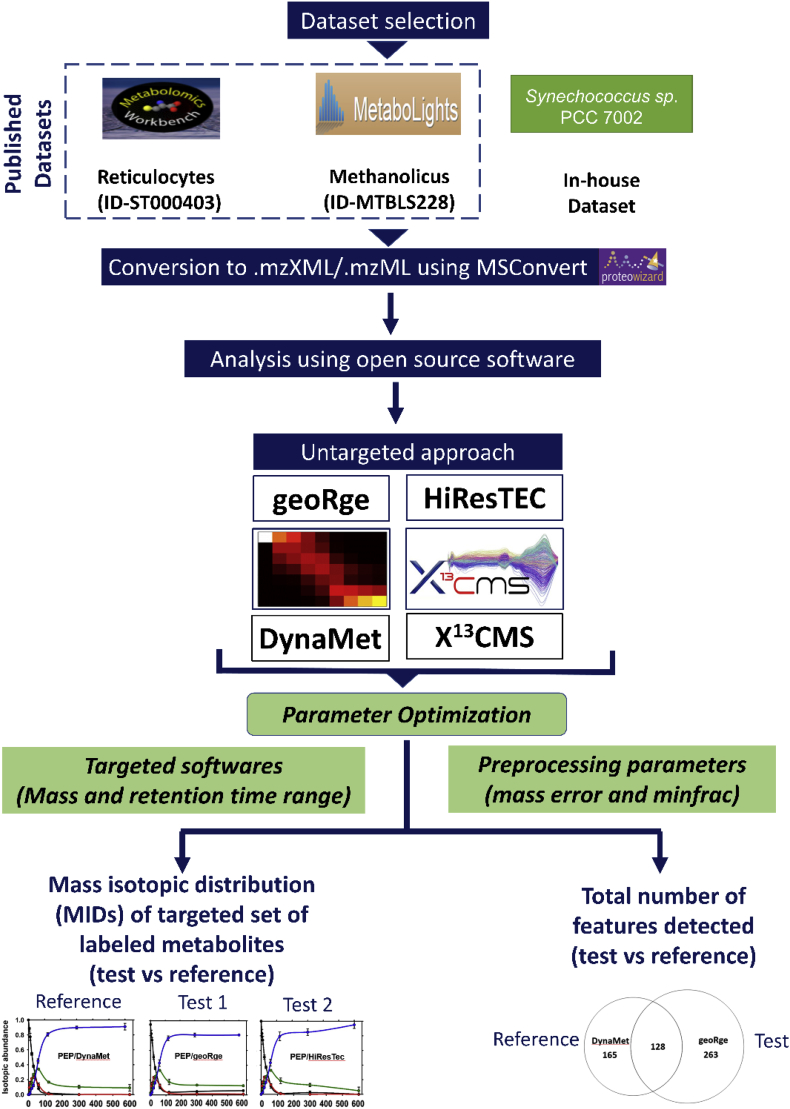
Workflow used in this study. Three datasets were selected, two published (‘Methanolicus’ and ‘Reticulocytes’) and one generated in-house (*Synechococcus* sp.). Software tools DynaMet, X^13^CMS, geoRge and HiResTEC were used for untargeted MID analysis of the 3 datasets and the total features detected were compared between them. A targeted MID analysis was also carried out with all three datasets, using benchmarked metabolites obtained with the ‘reference’ software (DynaMet for the Methanolicus dataset and mzMatch-ISO for the Reticulocyte dataset) and subjected to reanalysis by one or more ‘test’ software. In case of the in-house dataset, the vendor provided software tool MultiQuant^TM^ was considered the ‘reference’.

The purpose of this study was manifold: (i) to test the ability of freely available software in detecting isotopic enrichment in metabolites in an untargeted manner, (ii) compare results from different software, and (iii) assess the ease of use of these tools from the user’s perspective. The workflow adopted for this study has been summarized in Fig. 1. We chose to test the software tools, X^13^CMS (Patti and CMS, 2014), geoRge (Capellades et al., 2016), DynaMet (Kiefer et al., 2015) and HiResTEC (Hoffmann et al., 2018) to quantify ^13^C enrichment in intracellular metabolites from HR-LC/MS data derived from three independent studies that were diverse in the biological systems, LC methods and MS hardware used (Table S1). Despite the applicability of these software tools for ^13^C-MFA, very few cited studies could be found that have implemented them for this purpose.

Three separate ^13^C labeled LC/MS datasets were selected, two available in the public domain (Reticulocytes (Srivastava et al., 2017) and Methanolicus (Kiefer et al., 2015)) and one generated in-house (*Synechococcus* sp.). For the public domain datasets, a curated list of the m/z features that accumulate isotopic ^13^C was available from the respective publications. From this, metabolites pertaining to the central carbon pathway were selected. Reference software for a particular dataset detected a subset of these metabolites (referred to as the benchmark list) and were used to compare the output from the ‘test’ software. For example, the ‘Reticulocytes dataset’ was reanalyzed using the test software tools X^13^CMS (Patti and CMS, 2014), geoRge (Capellades et al., 2016) and HiResTEC (Hoffmann et al., 2018). The data was then compared with the results obtained by the authors of the study through mzMatch-ISO (Chokkathukalam et al., 2013). DynaMet could not be used here as this tool incorporates a kinetic model and requires a detailed time course study. On the other hand, the ‘Methanolicus dataset’ was reanalyzed using X^13^CMS, geoRge and HiResTEC, and subsequently compared with data obtained using DynaMet. In case of the *Synechococcus* sp. data, the analyses by X^13^CMS, geoRge, DynaMet, and HiResTEC were compared to the vendor provided software, MultiQuant. This reference software could only be applied towards targeted comparisons as it does not support untargeted analysis. In this study, coverage of metabolites in the benchmarked lists for each dataset have been used (true positives) to provide a quantitative assessment of the performance of each software in untargeted analysis.

### 3.2 Overview of software tools used in this study

A number of software are currently available that claim to automate untargeted detection of isotopic label incorporation from HR-LC/MS data. Of the ones that are a part of this study, some key features such as post data acquisition workflows, statistical tests used, and ease of data visualization have been summarized in Table 1. These features affected critical aspects of analysis such as detection of features and their isotopologues, MID patterns, number of false positives and redundancies which in turn influenced the software performance across datasets. For instance, X^13^CMS (Patti and CMS, 2014), geoRge (Capellades et al., 2016), and HiResTEC (Hoffmann et al., 2018) use XCMS (Smith et al., 2006) to detect metabolite peaks and to perform retention time alignment. However, labeled feature detection in geoRge uses a comparison of potential isotopic peaks between labeled and unlabeled samples while X^13^CMS looks for features separated by m/z values corresponding to tracer mass difference within the same retention time window, in an iterative manner. As a result, geoRge yields overall fewer features and also a smaller number of false positives compared to X^13^CMS. In this study, features showing inconsistencies in labeling patterns or an unusual number of isotopologues have been considered as false positives. HiResTEC provides a further improvement, by reducing the redundancies and false positives through the use of a deconvolution algorithm that accesses the raw data and uses automated quality control steps to filter out the noise. In this study, the performance of DynaMet showed the largest variance compared to other test software possibly because of its distinctly different pre- and post-data processing algorithms. DynaMet uses the FeatureFinderMetabo application from OpenMS (Kenar et al., 2014) for the detection of peaks and m/z traces while for postprocessing, the “isotope_regrouper” algorithm is implemented (Kiefer et al., 2015). Additionally, DynaMet also uses first order fitting of labeling profiles to determine the kinetics. This allows the user to identify the compounds based on m/z value, through links to the KEGG database. It should also be noted that X^13^CMS can readily handle data from only two time points for analysis because of intrinsic limitations of this software. Therefore, when analysing multi-time point data used in this study (Methanolicus and *Synechococcus* sp. datasets), we have analysed only the initial and final time points using this software.

**Table 1:** Comparison of features of selected freely available software tools for processing high resolution mass spectrometry data for the untargeted quantification of ^13^C enrichment in metabolites.

Feature	X^13^CMS^a^	geoRge^a^	HiResTEC^a^	DynaMet^a^	mzMatch-ISO^a^
Platform	R	R	R	Python	R
Pre-processing	XCMS	XCMS	XCMS	OpenMS	XCMS
Statistical Analysis^b^	Y	Y	Y	N	Y
Time course kinetics	N	Y	Y	Y	Y
Metabolite Identification^c^	N	Y	N	Y	Y
MID Correction	Y	N	N	Y	N
**Ease of use**					
GUI	N	N	N	Y	N
Peak evaluation^d^	Y	N	Y	Y	Y
Reliance on previous Knowledge	Y	Y	Y	Y	Y
Automated Data Visualization^e^	N	N	Y	Y	Y
Output Format	.xslx, pdf	.xslx	.xslx, pdf	Python table file	.tsv/.pdf

a Y and N in the table indicate whether the feature is available or not available, respectively.

b Used for labeled vs unlabeled peak detection.

c geoRge and mzMatchISO provide limited in-built data for metabolite identification at MS1 level. DynaMet allows identification based on KEGG database.

d The Peak evaluation capability such as extracted ion chromatograph and peak areas in the software panel.

e Data visualization in terms of ^13^C enrichment plots and Mass isotopologue distribution plots.

Data output formats varied between the tested software. X^13^CMS generates a file in the pdf format which shows the ^13^C enrichment in all detected features. Numerical values for the intensities of the same features are provided separately in an Excel spreadsheet. geoRge, on the other hand, provides a single Excel spreadsheet showing the intensity data of all detected features, from which subsequent MIDs and ^13^C enrichment can be quantitated. HiResTEC provides the added advantage of Quality Control (QC) plots as a pdf file for visual examination of data quality and also allows correction of MIDs for natural abundance. DynaMet also provides natural abundance correction in MIDs and allows visualization of the labeling patterns of all detected feature as heat maps.

### 3.3 Parameter optimization

**Fig. 2:**
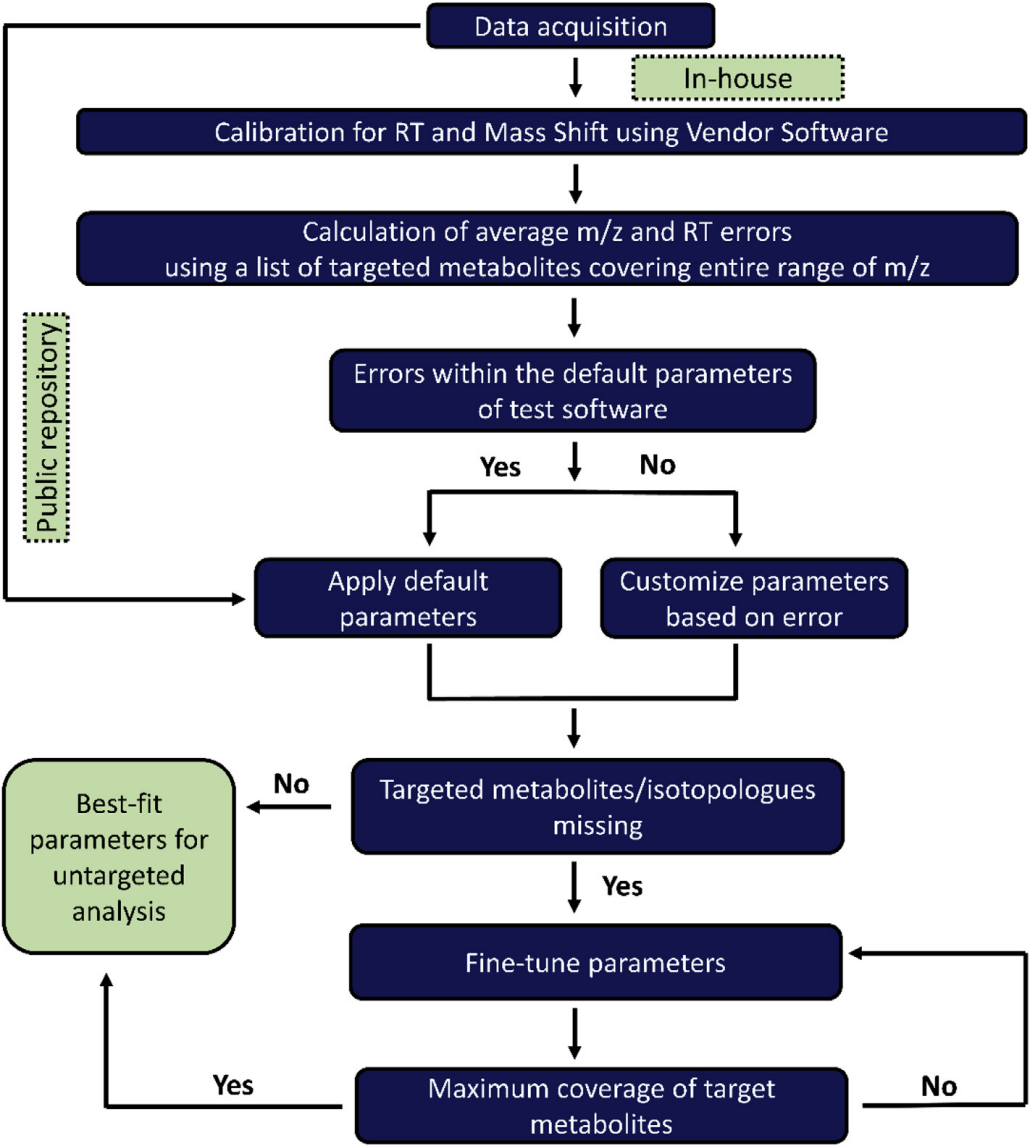
Flow chart representing the strategy used for optimizing parameters for the datasets used.

When analysing the different datasets used in this study, finding optimal parameters for each individual software was of critical importance. The parameters selected influenced the total number of metabolites and their isotopologues detected with each software. When optimizing parameters for a particular software, quantitative factors such as the total number of detected features and extent of true feature detection are often considered. In this study, the focus was on the untargeted analysis of three individual datasets and expectedly a major challenge faced was the absence of a list of true features with which to carry out quantitative evaluation such as Receiver Operator Characteristic (ROC). Such analyses would normally help in determining true and false positive rates for sensitivity and specificity of feature detection respectively. In lieu of this, a targeted approach was used to provide a quantitative framework for tuning parameters for each individual analysis (Fig. 2). First, a list of targeted set of metabolites was created for every individual dataset. This list was then used to evaluate the output from an individual software after different parameters were applied. For a particular dataset, the parameter settings used by the authors of each individual software was used as a starting point (default settings). When these were not sufficient to detect a majority of the benchmarked metabolites, a range of other values were tried till no further improvement in coverage could be achieved. Parameters for a test software was considered optimized when it ensured maximal coverage of the benchmarked list of metabolites and their isotopologues. These parameters were then subsequently applied to untargeted analysis using the same software. This general strategy could be applied by new users of these software tools and applied to any selected dataset.

The different MS platforms used for data acquisition often cause individual datasets to have their own mass errors and RT shifts. This is often due to several unavoidable reasons such as differences in the MS settings used during data acquisition, matrix effects, and the instrument drift. These have to be accounted for, requiring internal calibrations for individual datasets. This often necessitates the use of different parameters when analysed with different software. This can be seen in the list of optimized parameters used for each software of this study in Table 2. Also, the inherent algorithmic differences of each software made it imperative to fine-tune parameters for each individual software tool within a single dataset. The software tools geoRge, X^13^CMS and HiResTEC use an XCMS generated feature list, hence the XCMS parameters such as mass error (ppm) and minfrac were first fine-tuned to improve peak picking (Kenar et al., 2014). This led to an increase in the number of features and their isotopologues detected from all datasets. After optimizing the parameters of the pre-processing software, parameters specific to the software tools needed to be additionally optimized. In the case of geoRge, tuning of software specific parameters like basepeak mass error (ppm) and basepeak min intensity led to a further increase in total number of features detected. In DynaMet, tuning of the parameters did not show improvement for Synechococcus sp. dataset.

**Table 2:** Optimized parameters used for analysis using geoRge, X^13^CMS DynaMet, and HiResTEC.

Parameters		Synechococcus sp.	Reticulocytes	Methanolicus
Pre-processing XCMS	Mass error	25	5	5
	mzwid	0.05	0.015	0.015
	minfrac	0.1	0.1	0.4
geoRge	fc threshold	1.5	1.5	1.5
	p-value threshold	0.05	0.05	0.05
	PuInc limit	500	4000	500
	Basepeak mass error	25	15	15
	Basepeak min intensity	1000	2000	1000
X^13^CMS	RT win	10	10	10
	ppm	20	15	10
DynaMet	maxMzDifferencePairfinder	0.02	Not used^a^	0.01
	mz_diff	0.02	Not used^a^	0.005
	rt_diff	100	Not used^a^	100
	common_noise_threshold_int	600	Not used^a^	1000
	common_chrom_peak_snr	2	Not used^a^	3
	common_chrom_fwhm	25	Not used^a^	25
	mtd_mass_error_ppm	5	Not used^a^	15
	isolation width	0.01	Not used^a^	0.003
	max_nrmse	0.5	Not used^a^	0.8
	maxMzDifferencePairfinder	0.02	Not used^a^	0.01
HiResTEC	dmz	0.0125	0.0125	0.0125
	dRT	5	5	5

a DynaMet was not used for the reticulocuytes dataset as the software includes a kinetic modeling step that requires a time course study.

### 3.4 Qualitative comparison of results from different tools

**Fig. 3:**
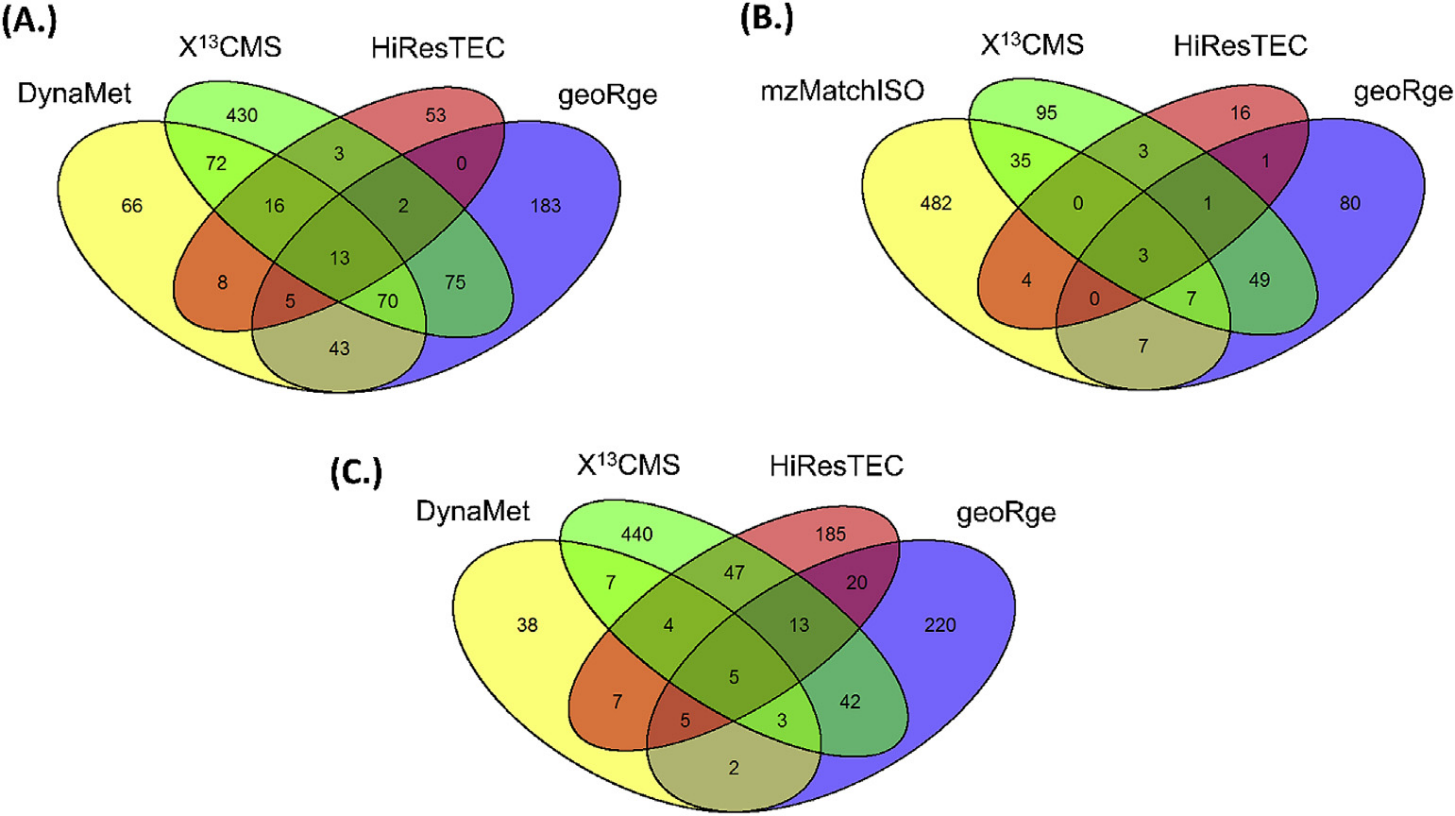
**Comparison of the total number of features detected in an untargeted analysis of a given dataset by different software tools.** Venn diagrams for (A) Methanolicus data, (B) Reticulocytes data and (C) *Synechococcus* sp. data.

After parameter optimization, we examined the number of features detected by a given software tool in an untargeted manner (Table 3) and the overlap of features between the different tools used for particular datasets (Fig. 3). Here, a feature implies an unique m/z in which ^13^C enrichment has been detected. It should be noted that the number of detected isotopologues or the MIDs obtained from the different software were not compared at this stage of analysis. In general, the tools detected a large number of features although the overlap between any two software tools was always less than 50% for the detected features. Among the tools, geoRge (Capellades et al., 2016) detected the highest number of features for the Methanolicus (Kiefer et al., 2015) and *Synechococcus* sp. datasets. Although the number of features detected by X^13^CMS was greater than geoRge for these datasets, it should be noted that this analysis was the output of only two time points (initial and final).

**Table 3:** Total number of features obtained from an untargeted analysis of the three datasets and percentage of features detected from the targeted analysis of benchmark metabolites.

Total number of features in untargeted analysis			
Software tool	Datasets		
	*Synechococcus* sp.	*Methanolicus*	*Reticulocytes*
geoRge	310	391	148
DynaMet	71	293	Not Used^a^
X^13^CMS	561^b^	681^b^	193
HiResTEC	286	100	28
True positives (%) detected in targeted analysis^c^			
Software tool	Datasets		
	*Synechococcus* sp.	*Methanolicus*	*Reticulocytes*
geoRge	61.1	68	33.3
DynaMet	22.2	100	Not Used^a^
X^13^CMS	66.6	81	66.6
HiResTEC	72	81	13.3

a DynaMet requires more than two time points for the analysis.

b Only initial and final time points have been used for the analysis as X^13^CMS can handle only two samples at a time.

c True positives based on benchmarked list of metabolites.

For the Methanolicus dataset, out of 293 labeled features obtained through the reference software DynaMet, about 44% could also be detected through geoRge while HiResTEC (Hoffmann et al., 2018) detected approximately 15% of the features (Fig. 3A). X^13^CMS on the other hand, showed an overlap of ~58% when considering just the initial and final time points. For the Reticulocytes dataset, the reference software mzMatch-ISO detected 538 features. Of these, 80 features (~8%) were common with X^13^CMS and only 17 features (~3%) were common with geoRge (Fig. 3B). HiResTEC however detected very few features from this dataset (28) with minimal overlap.In terms of features detected, no single tool performed the same across the datasets. Further, no two tools showed significant overlap between detected features in a particular dataset.

**Fig. 4:**
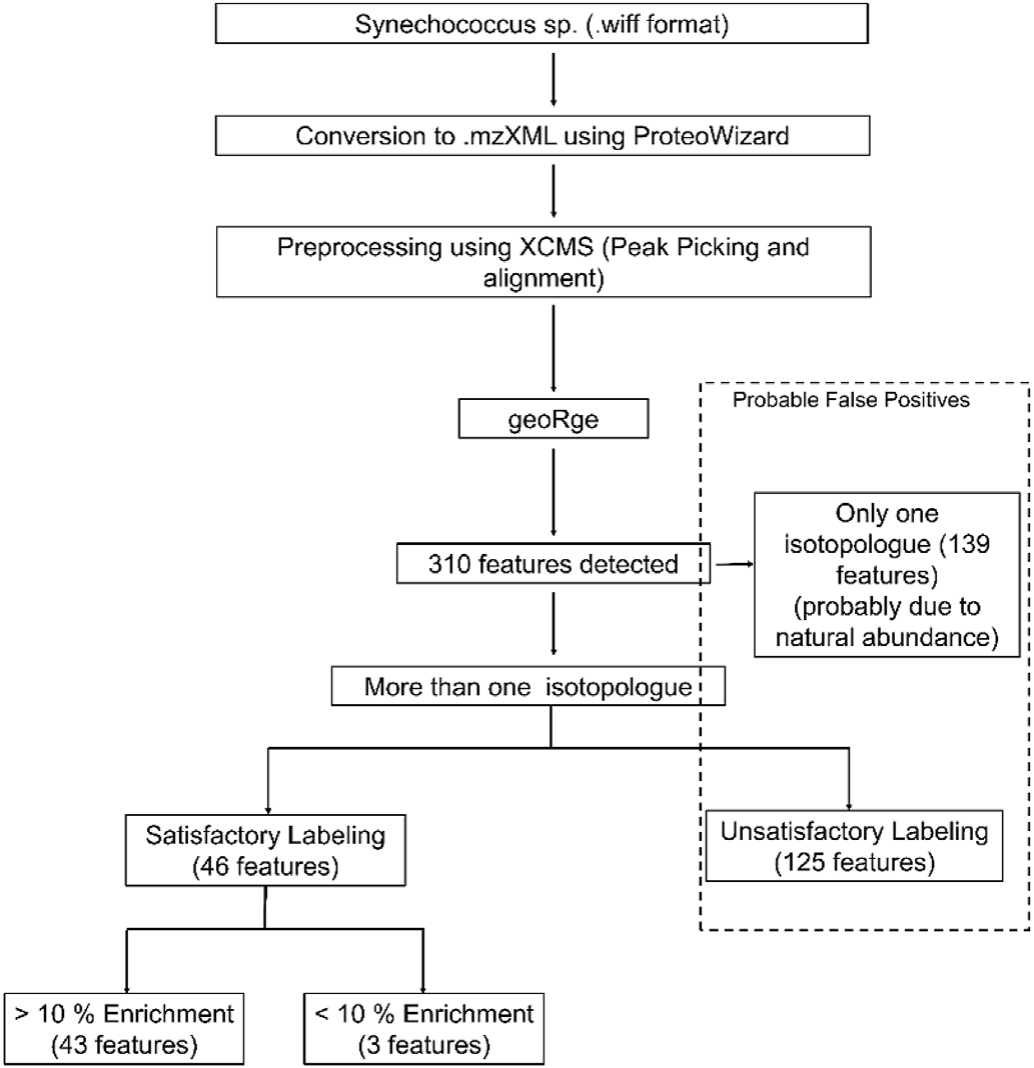
**Workflow for the untargeted analysis of the *Synechococcus* sp. dataset using geoRge.** False positives were removed on the basis of number of isotopologues detected for each feature and their respective labeling patterns. Satisfactory labeling refers to a gradual progression in the ^13^C enrichment while unsatisfactory labeling refers to unexpected progressions in ^13^C enrichment, conflicts in masses, and more than expected number of isotopologues.

In the *Synechococcus* sp. dataset, the test software X^13^CMS, geoRge, DynaMet, and HiResTEC were compared amongst themselves (Fig. 3C). The tool geoRge, provided 310 unique features with label incorporation, after elimination of obvious redundancies. Upon further removal of all probable false positives, only 46 features remained which showed satisfactory labeling patterns (Fig. S1, Table S2). The number of isotopologues and their Mass Isotopologue Distributions (MIDs) were taken into account for manual removal of probable false positives (Fig. 4). Of the features that were eliminated in this process, some showed the occurrence of only a single isotopologue possibly due to the natural abundance of ^13^C. Few others lacked a gradual progression in ^13^C enrichment over time. Such unexpected labeling patterns could be the result of potential artifacts, conflicts in isotopologues, and degeneracies (Table S3).

HiResTEC showed a similar range of feature detection for this dataset, providing 286 features with only ~15% of the features overlapping with geoRge. On the other hand, DynaMet which only allowed replicates to be analysed one at a time yielded varying number of features for each replicate revealing their inherent non-homogeneity. After the removal of redundant features, and merger of data from both replicates, DynaMet revealed 71 features in total. This was significantly less than geoRge and HiResTEC (Fig. 3C). Additionally, very few features showed satisfactory labeling patterns. In our experience, geoRge and HiResTEC performed better than other software tools in untargeted analyses of time-course datasets. X^13^CMS detected a total of 561 labeling features showing around 20% overlap with geoRge and HiResTEC. However, since only initial and final time points were used for this analysis, the detected features cannot be reliably compared with the other tools.

### 3.5 Comparison of MID from different tools

**Fig. 5:**
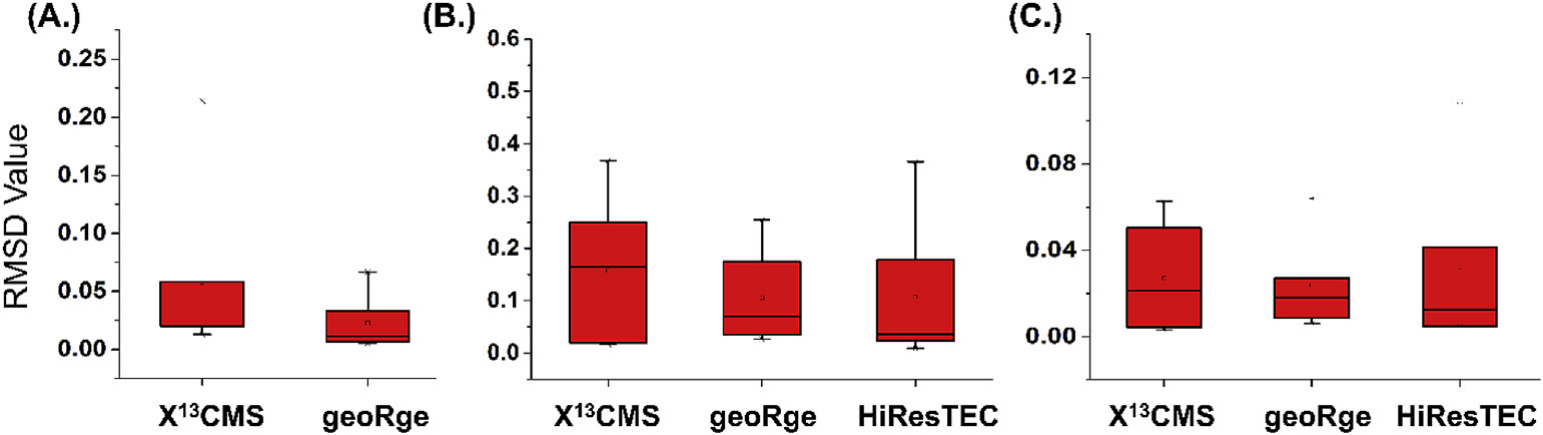
**Comparison of quantitated MIDs for benchmarked metabolites between different test software using Root Mean Square Deviation (RMSD)**. A.) The RMSD values for quantitated MIDs of benchmarked metabolites was calculated for ‘Reticulocytes’ dataset using the test software X^13^CMS and geoRge and MIDs obtained from mzMatch-ISO were considered as the reference. B.) The RMSD values for MIDs quantitated using test software X^13^CMS, geoRge and HiResTEC for ‘Methanolicus’ dataset in comparison to DynaMet as reference C.) The RMSD values for MIDs quantitated using X^13^CMS, geoRge, and HiResTEC for the *Synechococcus* sp. dataset in comparison to MultiQuant (reference).

For a software tool to be of use in ^13^C MFA, it should be able to reliably detect all the isotopologues for a detected metabolite and accurately obtain their MID values. A quantitative comparison of MID profiles was performed for 23 metabolites of interest belonging to the central carbon pathway (Table S4). Of these, MIDs were available for 18 metabolites using MultiQuant^TM^ on the *Synechococcus* sp. dataset, 16 metabolites using DynaMet on the Methanolicus dataset and 15 metabolites with the mzMatch-ISO-analysed reticulocytes dataset. This was then used as the gold standard to evaluate the test software for each dataset (Tables S5–S7) and Root Mean Square Deviation (RMSD) was calculated as a measure of accuracy of MIDs obtained using different tested software (Fig. 5, Table S8). RMSD was calculated for the MID values of the detected metabolites using equation (1). For the *Synechococcus* sp. dataset, DynaMet detected only 4 of the 18 metabolites that were quantified with the reference software MultiQuant and hence DynaMet was not included for comparison. For all three datasets, a greater distribution of RMSD values was observed for X^13^CMS followed by geoRge (Fig. 5).

(1) $$\text{RMSD}=\sqrt{\frac{\sum_{j=1}^{m}\sum_{i=0}^{n}{\left(y_{\mathrm{ij}}-x_{ij}\right)}^{2}}{m\times n}}$$

where x & y are the fractional abundances for an isotopologue provided by the reference and test software respectively, and m & n are the total number of time points and total number of isotopologues of a particular metabolite, respectively.

Based on the trends observed from the analysis of the three datasets, we categorized the detected features into two groups; group 1 features, which show a close match in the MIDs between reference and test software and group 2 features that show non-detection of some isotopologues or mismatches in MIDs. We could ascribe some of the deviations in group 2 features to the poor chromatographic separation, low abundance or variation in retention time (RT). A visual inspection of the peak quality was made for each metabolite during analysis.

#### 3.5.1 Group 1: Metabolites for which the quantitation of MIDs correlated between the reference software and at least one test software

**Fig. 6:**
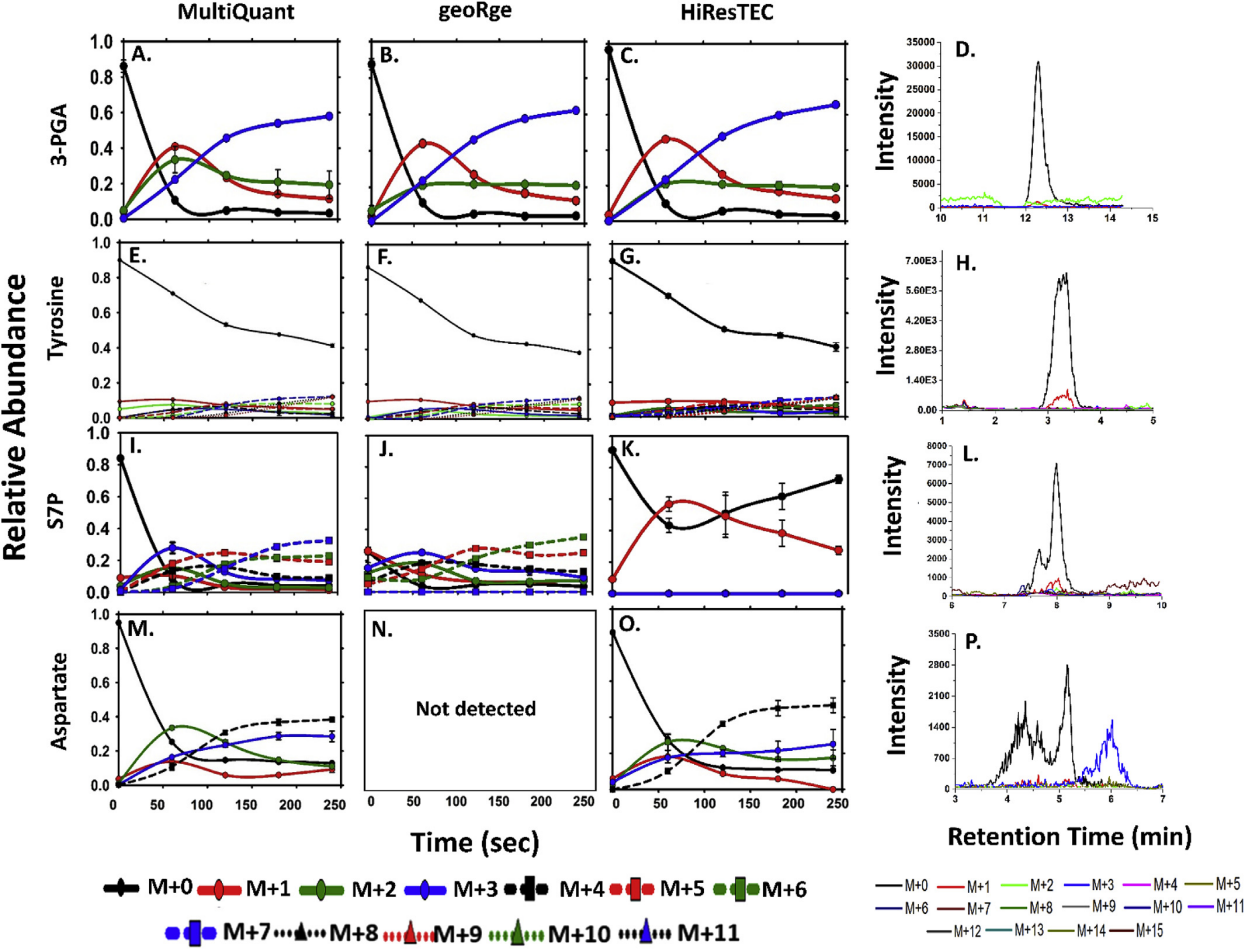
**Comparison of the dynamic labeling patterns of selected metabolites 3-PGA, Tyrosine, S7P and Aspartate for the *Synechococcus* sp. dataset.** These metabolites were identified and their MIDs quantitated using MultiQuant. The raw data was re-analysed using geoRge and HiResTEC. The XICs of all the relevant isotopologues of the metabolite for the t ​= ​0 ​min timepoint (the unlabeled sample), are shown to assess the peak quality and potential conflicts with isotopologues.

**Fig. 7:**
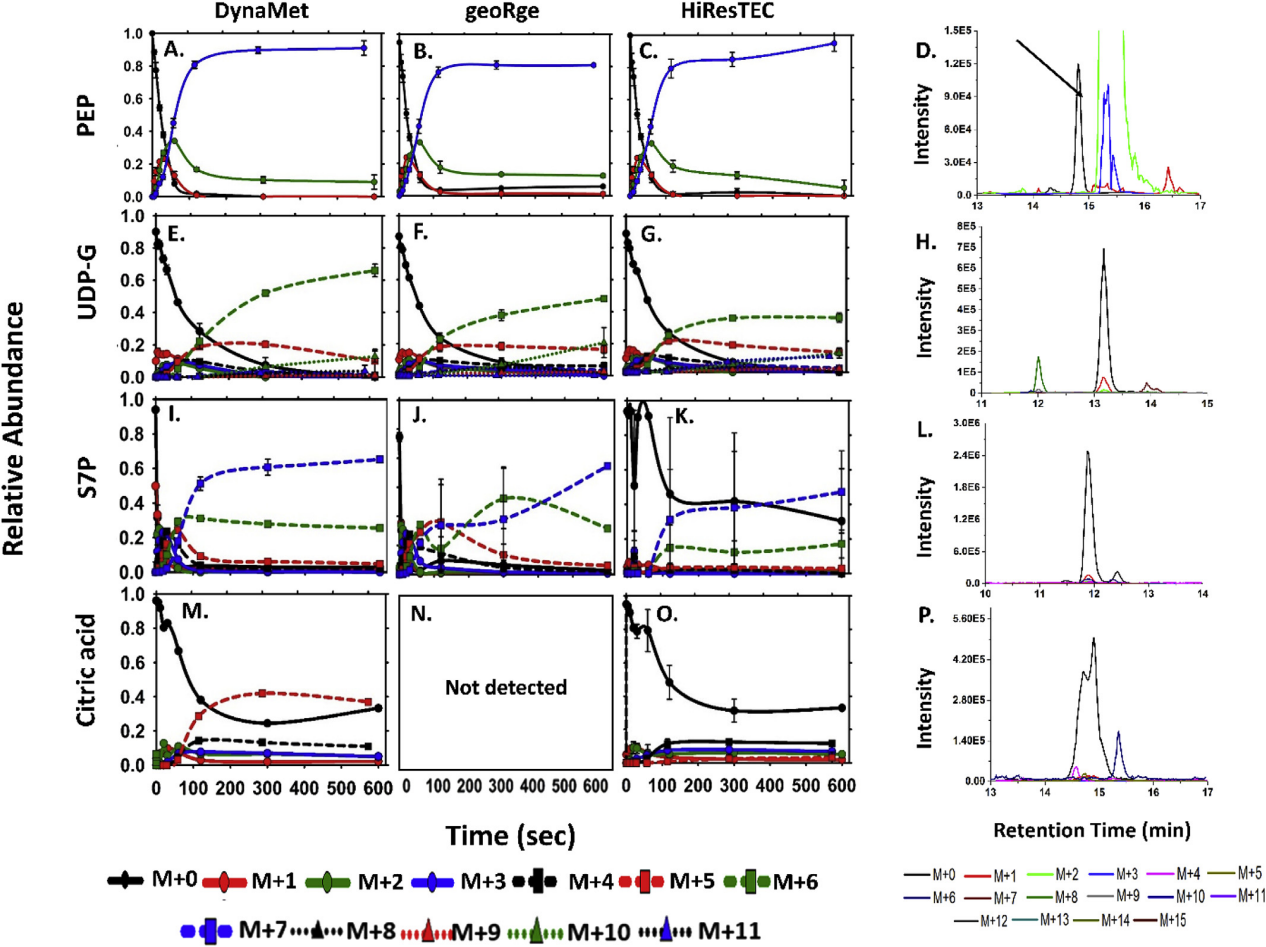
**Comparison of the experimentally measured MIDs of PEP, UDP-G, S7P and citric acid which were obtained through the targeted analysis of the ‘Methanolicus’ dataset.** The data for DynaMet has been taken from (Kiefer et al., 2015) and treated as reference software. The raw data was reanalyzed using geoRge and HiResTEC. The XICs of all the relevant isotopologues of the metabolite for the t ​= ​0 ​min timepoint (the unlabeled sample), are shown to assess the peak quality and potential conflicts with isotopologues. The arrow indicates the peak for PEP.

**Fig. 8:**
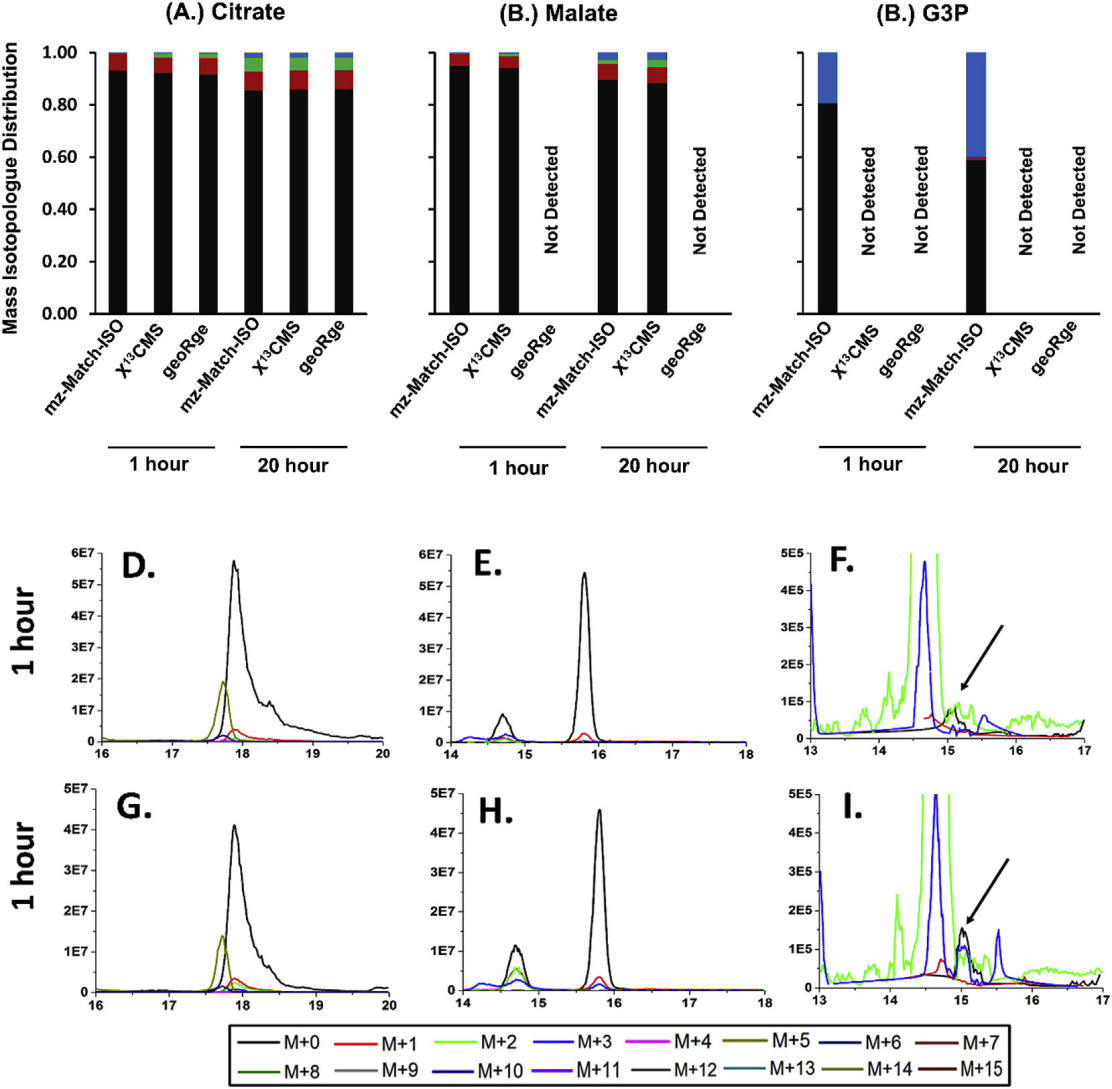
**Comparison of the dynamic labeling patterns of Citrate, Malate and G3P which were obtained through the targeted analysis of the ‘reticulocytes’ dataset.** The data for mzMatch-ISO has been taken from (Srivastava et al., 2017) and treated as reference software. The raw data was re-analysed using X^13^CMS and geoRge. The XICs of all the relevant isotopologues of the metabolites for two timepoints, t ​= ​0 ​h (unlabeled sample) and t ​= ​20 ​h (fully labeled sample), are both shown to assess the peak quality and potential conflicts with isotopologues. The arrow points to the peak for G3P.

For the metabolites used for comparison within a particular dataset, many showed similar labeling patterns between the reference and test software. For instance, for the *Synechococcus* sp. dataset, 3-Phosphoglyceric acid (3-PGA) (Fig. 6, A–C) and Glucose-6-phosphate (G6P) (Fig. S2, G-I) show a good match in labeling profiles across all tested software. Visual examination of the chromatographic data shows that many of these metabolites have good quality peaks as seen from the Extracted Ion Chromatogram (XIC) of 3-PGA (Fig. 6, D). A similar example can be seen in the ‘Methanolicus’ dataset with Uridine di-phosphate glucose (UDP-G), where a good peak shape correlates with well-matched MIDs between the reference and test software (Fig. 7, E–H). Other metabolites in this category include Phosphoenolpyruvate (PEP) despite the presence of a second closely-placed peak in the chromatogram (Fig. 7, A–D). Within the ‘Reticulocyte’ dataset, similar MID patterns across software are seen in the case of Citrate (Fig. 8, A) showing good peak quality for both time points (Fig. 8, D & G) and 3-PGA (Figs. S3 and C).

Amino acids such as tyrosine and glutamate, are often found to have a lower enrichment of the label in ^13^C samples because they are relatively farther away from the main node of label incorporation in the metabolic network. However, although both could be detected from the *Synechococcus* sp. dataset by geoRge and HiResTEC, the MIDs correlated with MultiQuant only in the case of tyrosine (Fig. 6, E–G) and not glutamate (Fig. S2, Y-AA). The peak quality of tyrosine was found to be distinctly better (Fig. 6, H) than that of glutamate (data not shown) suggesting that data quality might have had a role to play in this deviation.

#### 3.5.2 Group 2: Metabolites which could not be reliably detected or had non-correlated MIDs between reference and a majority of the test software

For each dataset used in this study, test software led to non-detection of some of the isotopologues or mismatched MID patterns for a significant number of the metabolites with respect to the reference software. Since geoRge was the only test software tool that could be used reliably across all datasets, we evaluated the percentage of its detected metabolites that showed a strong MID correlation with the reference software of each dataset. For instance, only 50% of the detected metabolites showed a correlation with the MIDs generated with MultiQuant for the *Synechococcus* sp. dataset. In case of Methanolicus and Reticulocytes, correlated MIDs could be seen for only 7% and 20% of the metabolites, respectively. It appears that the quality of the chromatographic peak (peak shape, intensity, shoulder/split peaks) and other qualitative aspects of the data such as mass conflicts, homogeneity among replicates may adversely affect accurate calculation of MIDs.

The concurrence of poor peak quality with non-correlated MID profiles, was seen for several metabolites in all three datasets. For instance, for Sedoheptulose-7-Phosphate (S7P) a low intensity, split peak is seen for the *Synechococcus* sp. dataset (Fig. 6, I–L). Spilt peaks can often be erroneously integrated, a possible reason for the mismatch seen with the reference MID profile. For the Reticulocytes dataset, UDP-G showed a poor peak quality across replicates while split peaks were noticed in glucose (Fig. S4). These factors might have contributed to the mismatched MID profiles seen for these metabolites.

Apart from the non-correlated MIDs, certain metabolites of interest were completely missed by the test software. Examples of this include Aspartate (Fig. 6, M–O) in *Synechococcus* sp. dataset (non-detection by geoRge), Glyceraldehyde-3-Phosphate (G3P) in the reticulocytes dataset (non-detection by both X^13^CMS and geoRge, Fig. 8, C, F, I), and Citrate for the Methanolicus dataset (non-detection by geoRge, Fig. 7, M–P). The issue of non-detection is commonly encountered when using geoRge, even if the peak quality is uncompromised. This could be attributed to lack of replicates (Ribulose-5-Phosphate, Fig. S5 D-F), lack of homogeneity amongst replicates (ADP: Fig. S5, M-O and 3-PGA: Fig. S5, AB AD) for the Methanolicus dataset and PEP (Fig. S3, H) in the Reticulocytes dataset or insufficient labeling (Malate in the Reticulocytes dataset, Fig. 8, B, E and H).

#### 3.5.3 User’s perspective on ease of use of the software

At the outset, each of the software tools used in this study imposed a substantial learning curve on the user. This included the need for navigation through the parameters and the vocabulary accompanying each software package. The optimization of parameters for automated analysis required multiple trials and manual checks. Apart from the extensive parameter tuning required, the dependence on the number of replicates used, as well as quality of chromatograms in the raw data affected downstream processing. To assess the ease-of-use of the software tools from the perspective of a new user, the following were considered: (i) Installation and operation (ii) Data input format (iii) Data output formats and (iv) Visualization of results. Some prior knowledge of software platforms and programming languages could help the first-time use of these software tools. X^13^CMS (Patti and CMS, 2014), geoRge (Capellades et al., 2016) and HiResTEC (Hoffmann et al., 2018) are R-based packages and also need the R-based XCMS package (Smith et al., 2006) for pre-processing. It should be noted that XCMS Online (TautenhahnRalf. et al., 2013), a widely used web-based package cannot be implemented for this set of software tools. Preliminary knowledge of R is required for the installation and usage of these packages for data analysis. Likewise, basic knowledge of Python is required while using DynaMet.

All the software tools accept data in the open formats of .mzXML or .mzML files. Raw HR LC/MS data can be easily converted to this format using ProteoWizard, which provides a user-friendly graphical interface. On the other hand, output of data from the test software are in different forms. geoRge provides the intensities of isotopologues for all features detected in all the samples at once, in the form of an Excel spreadsheet. This saves time while computing MIDs for eventual use in ^13^C-MFA. In case of DynaMet and HiResTEC, this information needs to be exported individually for each detected feature thereby making this process time-consuming. In terms of visualization of results, DynaMet does provide a representation of MID profiles as heat maps, a very useful tool for the quick visualization of labeling profiles. The user-friendly graphical interface of DynaMet also helps in the easy input of parameters and other operations. Likewise, X^13^CMS and HiResTEC provide ^13^C enrichment plots for detected features. Some software tools like HiResTEC, and DynaMet allow the user to visualize peak integration. This helps in the assessment of peak quality for individual detected features.

#### 3.5.4 Recommendations for new users and future software developers

For new users, selection of any one of the tested software tools will depend on the overall objective and experimental design of their studies. In labeling experiments, the number of time points being considered will significantly influence software choice. For instance, X^13^CMS can be used for experiments where only two time points are considered (unlabeled and labeled). On the other hand, geoRge, DynaMet and HiResTEC can be used for time-course experiments that aim to capture transient labeling for studies aimed at metabolic flux analysis. Furthermore, X^13^CMS implements statistics at a later stage of processing compared to geoRge and HiResTEC and this may affect the quality of results (Capellades et al., 2016). Annotation of the features detected through untargeted metabolomics is often a challenge. While several stand-alone tools are available that can perform metabolite identification like MS-DIAL (Tsugawa et al., 2015) and MetDIA (Li et al., 2016), geoRge and DynaMet can provide putative identification of labeled features through m/z matches with the Human Metabolome Database (HMDB) and Kyoto Encyclopedia of Genes and Genomes (KEGG) database respectively. Additionally, geoRge also allows the use of custom libraries in place of HMDB. Note that this identification is based on MS1 matches and needs to be validated by annotation with MS2 data by using other software.

For any selected tool, optimization of parameters is a key step to ensure the reliable detection of features in an untargeted analysis. As shown in this study, a targeted validation using a list of benchmarked metabolites can be used to test the suitability of selected parameters for the dataset being analysed. Initially, parameters used by the authors of the software can be used as default. These can subsequently be fine-tuned to obtain maximum coverage of metabolites and their isotopologues from benchmarked lists. The labeled features detected with optimized parameters can then be considered for downstream analysis. Additionally, data from some software tools like geoRge and DynaMet require manual curation to remove redundancies. HiResTEC on the other hand allows the removal of any such redundancies saving the user additional steps in analysis.

From our experience, analysis with the tested tools revealed some common challenges. We have faced issues with (i) non-detection of some of benchmarked metabolites in automated analysis despite satisfactory peak shape and intensity (ii) missing or disproportionate number of isotopologues for known metabolites (iii) presence of redundant features (iv) inability to detect labeling in fragments (MS2) in an untargeted manner and (v) lack of annotation for a significant fraction of the detected features. Future developers of software tools could aim to provide features that address some of these challenges.

## 4 Conclusion

For ^13^C Metabolic Flux Analysis, the precision that is required in MID quantitation poses a major challenge in analysing large datasets. To fully harness the available HR-LC/MS data in a high throughput, untargeted manner, a significant degree of automation is needed in the analysis process. Therefore, for a ^13^C-labeled dataset, selection of software tools that allow automated quantification of MIDs for a wide variety of metabolites is key. In this study, we have tested X^13^CMS, DynaMet, geoRge and HiResTEC, for their ability to detect and quantify MIDs of metabolites for three diverse LC/MS datasets. Software performance was gauged on the basis of (i) detection of m/z features that show ^13^C enrichment and (ii) accurate quantitation of MIDs. Although each software could quantify MIDs of a large number of metabolites, the results from the different software tools correlated only to a limited extent, both in terms of the number of metabolites detected and the labeling profiles. In addition, the test software lacked both sensitivity and selectivity, as seen from the large number of potential false positives that were detected in comparison to the reference software data. The MID values deviated between the test and reference software for a number of metabolites especially when the peak shape and signal to noise ratios were less than optimal.

Overall, geoRge and HiResTEC performed better than the other tools with all the datasets tested in this study with the former providing a facile data export option. This was gauged on the basis of consistent high numbers of metabolites detected across datasets in the untargeted analyses. In the targeted validation attempts, these software tools showed satisfactory labeling patterns for the majority of the detected metabolites and their isotopologues and less errors in MID quantitation as evident from the RMSD values. Although a number of false positives and redundant base peaks often result from the use of geoRge, the data can be readily curated. Fewer redundant base peaks were observed with HiResTEC, which implements a heuristic test to identify false positives. Despite the limitations and the learning curve involved, the software tools tested in this study provide the much-needed automation in the untargeted quantification of ^13^C enrichment and guidance for any further analysis. Note that these tools work with MS1 data leaving a big gap in untargeted analysis of ^13^C enrichment of fragments. Such data can be acquired in an untargeted manner using data independent acquisition (DIA) technique of tandem MS (Jaiswal et al., 2018). Tools that can detect ^13^C enrichment in an untargeted manner in the features from MS2 data can be a big boost to the analysis of positional labeling and will result in better resolution of the flux map. To address this and other challenges currently faced, future developers should focus on new tools that are user friendly, provide simplified parameterization and greater reliability of data analysis through reduction of noise and false positives.

## Author contributions

PPW conceived and supervised the research and acquired the funding. MCD, VM, DJ, CBP, and MSM performed the research., MCD, VM, and BM analysed the data and wrote the manuscript. BM, and PPW critically reviewed and edited the manuscript and prepared the final draft.
