# Supplementary material for: Evaluation of freely available software tools for untargeted quantification of 13C isotopic enrichment in cellular metabolome from HR-LC/MS data
Source: Metab Eng Commun. 2019 Dec 26;10:e00120. doi: 10.1016/j.mec.2019.e00120 (PMC6940703; doi:10.1016/j.mec.2019.e00120)
Supplement: Multimedia component 1 [file mmc1.pdf]

Supplementary Information

Evaluation of Freely Available Software Tools for Untargeted Quantification of ^{13}C Isotopic Enrichment in Cellular Metabolome from HR-LC/MS Data

Manohar C. Dange[a,†], Vivek Mishra[a,†], Bratati Mukherjee[a,b], Damini Jaiswal[a], Murtaza S. Merchant[a], Charulata B. Prasannan[a,b] and Pramod. P. Wangikar[a,b,c*]

[a]Department of Chemical Engineering, Indian Institute of Technology Bombay, Powai, Mumbai 400076, Mumbai, India.

[b]DBT-Pan IIT Center for Bioenergy, Indian Institute of Technology Bombay, Powai, Mumbai 400076, India, [c]Wadhwani Research Center for Bioengineering, Indian Institute of Technology Bombay, Powai, Mumbai 400076, India

†These authors contributed equally

*Address correspondence to PPW, Email: wangikar@iitb.ac.in; phone: + 912225767232

Table S1: Data acquisition and other details for the three datasets used in this study

S. No.	Name of Dataset/Reference/ Data repository	Biological system	LC platform	Chromatographic platform	Time course	Number of biological replicates	Number of technical replicates
1	'In-House'/Metabolomics workbench: doi: 10.21228/M87384	Cyanobacterial strain *Synecococcus sp.* PCC 7002	UHPLC (Shimadzu, Nexera LC-30 AD, Singapore) with C-18 synergi-hydro RP column (Phenomenex, Torrance, CA). The instrument was operated in negative ion mode for detection of metabolites and the corresponding fragments.	Triple TOF 5600+ mass spectrophotometer (SCIEX, Framingham, MA)	Yes	1	2
2	'Reticulocytes'/ (Srivastava et al.,2017)/MetaboLights repository: (study ID – ST000403, project ID – PR000315)	Human CD34+ Stem-Cell-Derived Reticulocytes	Dionex RSLC3000 UHPLC (Thermo) with ZIC-p (HILIC) column	Q-Exactive Orbitrap MS (Thermo) operating in rapid switch- ing positive (4 kV) and negative (−3.5 kV) mode electrospray ionization (capillary	Yes	3	N/A
3	'Methanolicus'/(Kiefer et al., 2015)/MetaboLights repository: (ID – MTBLS228)	*Bacillus methanolicus* MGA3	nanoscale ion-pair reversed-phase HPLC	LTQ-Orbitrap instrument operating in negative FT mode	Yes	2	N/A

Table S2: Total number of features detected in the Synechococcus sp. dataset, using geoRge in an untargeted manner. Only those features that showed satisfactory changes in isotopologue distributions over time, upon visual examination of labelling patterns, were included in this list.

S.No.	m/z	RT (min)	Putative ID	Difference between final and initial % enrichment
1	117.01	11.32	Succinate	13.79
2	133.04	9.32	Unknown	50.93
3	166.96	13.29	PEP	71.98
4	170.99	8.10	Glycerol-3-Phosphate	41.51
5	175.05	13.47	3-IPM	74.31
6	180.05	3.24	Tyrosine	17.28
7	184.97	12.34	3-PGA	81.13
8	205.02	12.31	Unknown	11.76
9	245.10	9.39	Unknown	18.90
10	254.97	11.28	Unknown	11.41
11	259.00	7.33	Unknown	50.91
12	259.00	7.94	G6P	51.59
13	276.97	12.20	Unknown	42.18
14	282.93	12.23	Unknown	71.40
15	289.01	7.94	S7P	62.79
16	315.08	4.40	Unknown	35.95
17	315.08	5.05	Unknown	14.07
18	323.01	10.42	UMP	4.63
19	324.03	10.42	Unknown	54.68
20	331.26	18.62	Unknown	6.62
21	334.93	13.21	Dimer of PEP	58.57
22	337.51	14.60	Unknown	35.34
23	347.01	10.69	Unknown	44.66
24	370.95	12.25	Dimer of 3PGA	71.65
25	391.00	12.30	Unknown	70.40
26	402.18	13.20	Unknown	64.44
27	462.95	12.24	Unknown	54.13
28	463.99	12.23	Unknown	50.82
29	505.95	14.87	ATP	15.89
30	524.59	14.94	Unknown	34.80
31	549.26	14.49	Unknown	5.01
32	554.99	12.21	Unknown	57.15
33	579.04	14.58	UDP-Glucoronate	36.60
34	587.22	5.06	Unknown	33.08
35	588.04	12.47	ADP-G	50.15
36	595.63	14.95	Unknown	62.48
37	596.13	14.95	Unknown	53.67
38	606.04	11.69	UDP-Nac Glucosamine	33.33
39	629.02	14.58	Unknown	40.48
40	676.10	14.68	Unknown	35.29
41	678.05	14.63	Unknown	37.64
42	683.24	1.56	Unknown	11.13
43	807.74	5.08	Unknown	32.71

Table S2 (contd.)

S.No.	m/z	RT (min)	Putative ID	Difference between final and initial % enrichment
44	808.14	15.36	Unknown	40.25
45	881.3419	5.077692	Unknown	38.29
46	915.204	7.627092	Unknown	35.12

Table S3: Remaining features from table S2. These features showed unexpected labelling patterns over time.

S. No.	m/z	RT	S.No.	m/z	RT
1	130.08	2.30	46	607.42	17.02
2	199.16	18.01	47	608.41	18.12
3	244.94	13.23	48	609.43	18.18
4	255.22	19.35	49	609.54	17.58
5	331.28	18.98	50	611.28	19.28
6	335.08	1.57	51	614.51	18.98
7	335.22	17.12	52	614.95	24.17
8	341.09	1.56	53	617.32	14.37
9	348.15	12.26	54	621.51	18.22
10	354.04	3.26	55	623.44	14.42
11	363.01	12.59	56	627.04	1.29
12	369.23	10.73	57	636.42	17.02
13	394.96	0.93	58	643.32	14.51
14	401.14	1.56	59	646.96	1.09
15	438.56	14.97	60	648.43	17.01
16	444.22	20.49	61	649.02	1.04
17	476.95	0.95	62	650.44	17.02
18	478.93	0.92	63	655.10	8.77
19	482.93	14.72	64	665.05	1.06
20	483.15	1.56	65	666.95	1.36
21	485.26	16.09	66	669.02	1.42
22	500.33	14.43	67	671.01	1.11
23	513.27	23.33	68	684.96	0.95
24	515.20	14.54	69	687.05	1.18
25	527.32	14.43	70	688.05	1.23
26	532.99	24.08	71	691.22	7.03
27	542.97	1.01	72	709.04	1.24
28	547.93	24.15	73	710.04	1.28
29	551.05	1.29	74	726.44	14.50
30	555.21	17.01	75	731.02	1.03
31	559.14	1.58	76	735.55	17.02
32	564.97	1.08	77	737.48	15.91
33	578.92	0.93	78	740.94	12.22
34	581.05	14.60	79	741.37	14.50
35	582.95	1.36	80	753.47	15.92
36	591.02	1.10	81	765.25	1.56
37	598.97	24.16	82	780.54	23.34
38	601.00	0.98	83	783.56	22.84
39	602.97	0.95	84	789.46	18.12
40	603.98	0.95	85	790.57	21.15
41	604.41	17.03	86	791.05	1.25
42	605.04	1.20	87	797.00	1.29
43	606.41	17.03	88	813.04	1.33
44	607.01	1.04	89	825.21	1.57
45	607.22	15.00	90	834.99	1.36

Table S3 (contd.)

S.No.	m/z	RT
91	835.01	1.13
92	838.00	1.29
93	841.25	1.58
94	845.99	1.32
95	846.06	1.33
96	857.01	1.21
97	873.05	1.25
98	875.99	1.28
99	876.06	1.27
100	884.05	1.33
101	886.99	1.32
102	890.59	15.87
103	891.59	15.85
104	894.97	1.34
105	895.48	1.34
106	898.01	1.21
107	899.01	1.21
108	908.68	17.84
109	909.00	1.25
110	909.50	1.25
111	910.95	1.36
112	916.21	7.68
113	917.01	1.19
114	919.71	18.00
115	920.00	1.29
116	920.50	1.29
117	924.79	20.47
118	927.99	1.32
119	928.49	1.32
120	933.73	18.19
121	934.73	18.19
122	939.01	1.21
123	950.50	1.25
124	959.02	1.09
125	970.52	1.19

Table S4: List of metabolites selected for a targeted detection and quantitation of MIDs, within the three datasets used in this study. These were used by the reference software tool assigned to each dataset to generate a 'benchmark list' against which certain test software were evaluated. This list includes metabolites commonly used in ^{13}C MFA.

Class of Metabolite	Metabolites Selected
Amino acid	Aspartate
	Glutamate
	Leucine
	Tyrosine
Carbohydrate	Sucrose
	Glucose
Sugar Phosphate	3-Phosphoglycerate (3PG)
	Glucose 6-Phosphate (G6P)
	Sedoheptulose 7-Phosphate (S7P)
	Ribose 5-Phosphate (R5P)
	Glyceraldehyde 3-Phosphate (G3P)
	Erythrose 4-Phosphate (E4P)
	Ribulose 5-Phosphate (Ru5P)
Organic acid	Succinate
	Malate
	Citrate
	Pyruvate
	Glycerate
	Fumarate
	Phosphoenolpyruvate
Others	UDP glucose
	ADP glucose
	Acetyl Co-A
	Uridine monophosphate (UMP)
	Adenosine diphosphate (ADP)
	Adenosine triphosphate (ATP)
	Nicotinamide-adenine dinucleotide (NAD)
	Fructose 1,6-bisphosphate (FBP)
	6-Phosphogluconate
	Shikimate

Table S5: Comparative evaluation of three test software tools, geoRge and HiResTEC in detecting and quantifying MIDs of benchmarked metabolites, within the in-house dataset, *Synechococcus* sp. Benchmarking was done with metabolites that were successfully detected and MID values manually quantified, using the reference software, MultiQuant. Metabolites with correlating MID patterns, across all software tools used for this dataset, have been marked with an asterisk.

**Class of metabolite**	**Metabolite**	**Reference Software[a]**	**Test Software[a]**	
		**Multiquant**	**geoRge**	**HiResTEC**
Amino Acid	Aspartate	✓	-	++
	Glutamate	✓	++	++
	Leucine*	✓	++	++
	Tyrosine*	✓	++	++
Carbohydrate	Sucrose*	✓	++	++
Sugar Phosphate	3-PGA*	✓	++	++
	G6P*	✓	++	++
	S7P	✓	+	+
Organic acids	Succinate	✓	-	++
	Malate	✓	++	+
	Citrate	✓	-	**++**
Others	UDP glucose	✓	-	**+**
	ADP glucose	✓	+	**+**
	Acetyl CoA	✓	-	+
	UMP*	✓	++	++
	ADP	✓	-	+
	ATP*	✓	++	++
	NAD	✓	-	-

[a] "✓" indicates the metabolites with quantitated MIDs generated by the reference software. Metabolites that could not be detected are indicated with a "-". Metabolites detected but with mismatch MID values or missing isotopologues indicated with "+". Metabolites with MIDs values in agreement with reference software are indicated with "++"

[b] Only initial and final time points were used for the analysis

Table S6: The performance of the test software**,** geoRge and HiResTEC, in the detection and quantification of MIDs of the benchmarked metabolites, generated from the Methanolicus dataset, using DynaMet. Metabolites with correlating MID patterns, across all software tools used for this dataset, have been marked with an asterisk

**Class of metabolite**	**Metabolite**	**Reference Software[a]**	**Test Software[a]**	
		**DynaMet**	**geoRge**	**HiResTEC**
Sugar Phosphate	3-PGA	✓	-	++
	G6P	✓	+	+
	S7P	✓	+	+
	R5P	✓	-	++
	G3P	✓	+	+
	E4P	✓	+	+
	Ru5P	✓	-	+
Organic acid	Citrate	✓	-	+
	PEP*	✓	++	++
Others	UDP glucose	✓	++	+
	Acetyl CoA*	✓	++	++
	ADP	✓	-	++
	ATP	✓	-	++
	FBP	✓	+	++
	6-Phosphogluconate	✓	-	+
	Shikimate	✓	-	-

[a & b] Refer to footnotes to Table S2

Table S7:. Results obtained from the **i**mplementation of $X^{13}CMS$ and geoRge as test software, for the benchmark list generated by mzMatch-ISO, using the Reticulocytes dataset. Metabolites with correlating MID patterns, across all software tools used for this dataset, have been marked with an asterisk.

**Class of metabolite**	**Metabolite**	**Reference Software[a]**	**Test Software[a]**	
		**mzMatch-ISO**	**$X^{13}CMS$**	**geoRge**
Carbohydrate	Glucose	✓	+	-
Sugar Phosphate	3-PGA*	✓	++	++
	S7P	✓	+	+
	R5P	✓	-	-
	G3P	✓	-	-
Organic acid	Succinate	✓	-	-
	Malate	✓	+	-
	Citrate	✓	+	+
	Pyruvate	✓	+	-
	Glycerate	✓	-	-
	Fumarate	✓	-	-
	PEP	✓	++	-
Other	UDP glucose	✓	+	+
	FBP	✓	+	-
	6-Phosphogluconate	✓	+	+

[a] Refer to footnotes to Table S2

Table S8: RMSD values for metabolites with matching MID profiles across all test software, for each of the individual datasets used in this study. MID values obtained from the respective reference software, were used for each calculation.

**Dataset and reference**	**Metabolite**	**Test Software**	
**In-house dataset analyzed with MultiQuant**		**geoRge**	**HiResTEC**
	**Time Points (seconds)**	**0, 60, 120, 180, 240**	**0, 60, 120, 180, 240**
	Leucine	0.070, 0.067, 0.051, 0.066, 0.063	0.019, 0.050, 0.028, 0.039, 0.057
	Tyrosine	0.034, 0.030, 0.023, 0.023, 0.0214	0.004, 0.010, 0.016, 0.014, 0.011
	Sucrose	0.005, 0.005, 0.007, 0.005, 0.007	0.002, 0.001, 0.006, 0.003, 0.008
	3-PGA	0.013, 0.032, 0.029, 0.038, 0.038	0.069, 0.038, 0.032, 0.050, 0.056
	UMP	0.007, 0.003, 0.003, 0.006, 0.012	0.011, 0.003, 0.006, 0.008, 0.010
	ATP	0.010, 0.006, 0.007, 0.008, 0.011	0.007, 0.002, 0.003, 0.003, 0.007
**Reticulocytes Dataset analyzed with mzMatch-ISO**		**X^{13}CMS**	**geoRge**
	**Time Points (hours)**	**1, 20**	**1, 20**
	3-PG	0.024, 0.015	0.030, 0.036
	UDP glucose	0.082, 0.009	0.004, 0.006
**Methanolicus Dataset analyzed with DynaMet**		**geoRge**	**HiResTEC**
	**Time Points (seconds)**	**0, 5, 10, 20, 30, 60, 120, 300, 600**	**0, 5, 10, 20, 30, 60, 120, 300, 600**
	PEP	0.031, 0.035, 0.021, 0.022, 0.023 0.019, 0.025, 0.052, 0.061	0, 0.030, 0.019, 0.022, 0.025, 0.020, 0.011, 0.029, 0.028

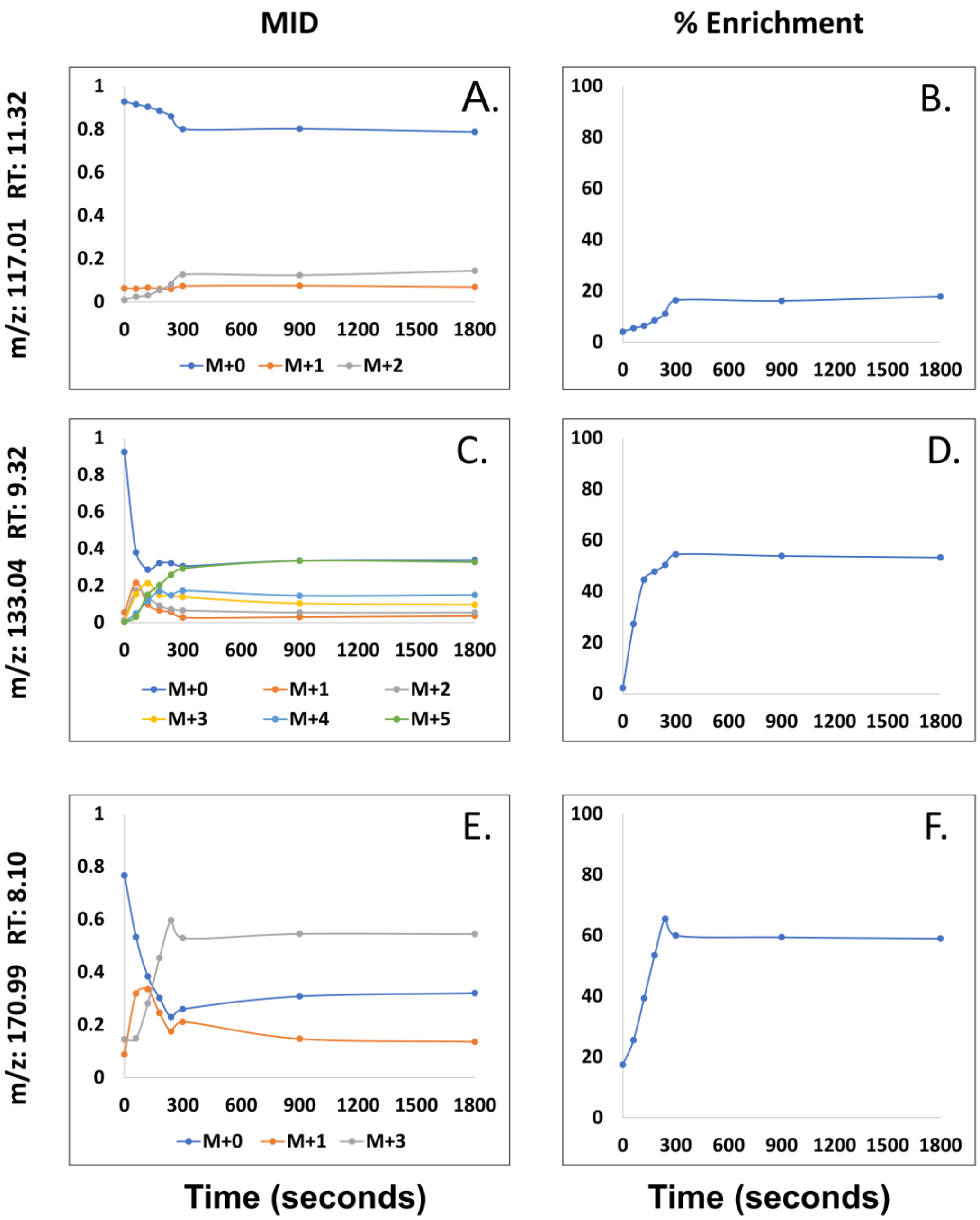

Figure S1: Labeling patterns for features which show satisfactory profiles over the time detected from the Synechococcus sp. dataset using geoRge in an untargeted manner. The m/z and RT of each individual feature are shown.

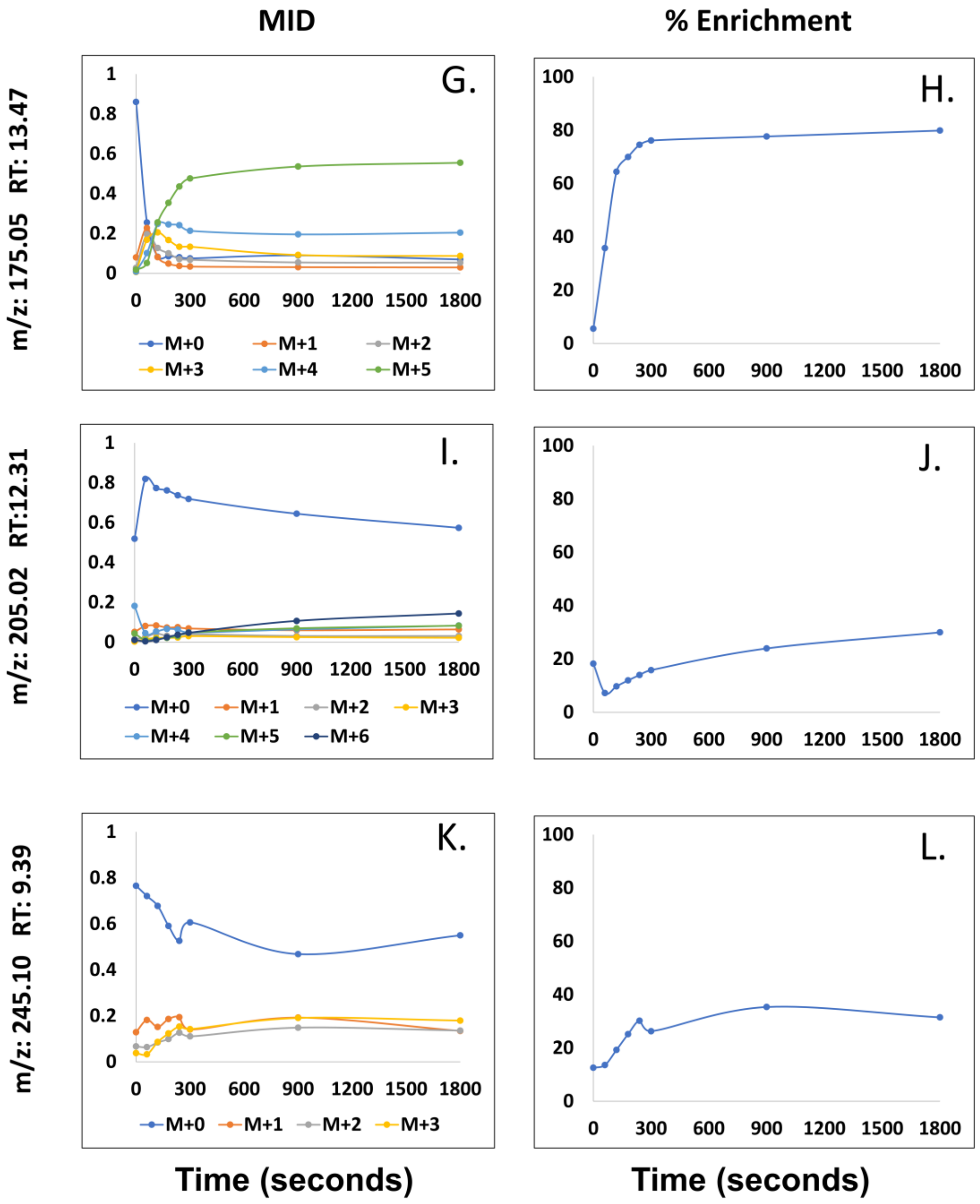

Figure S1 (contd.)

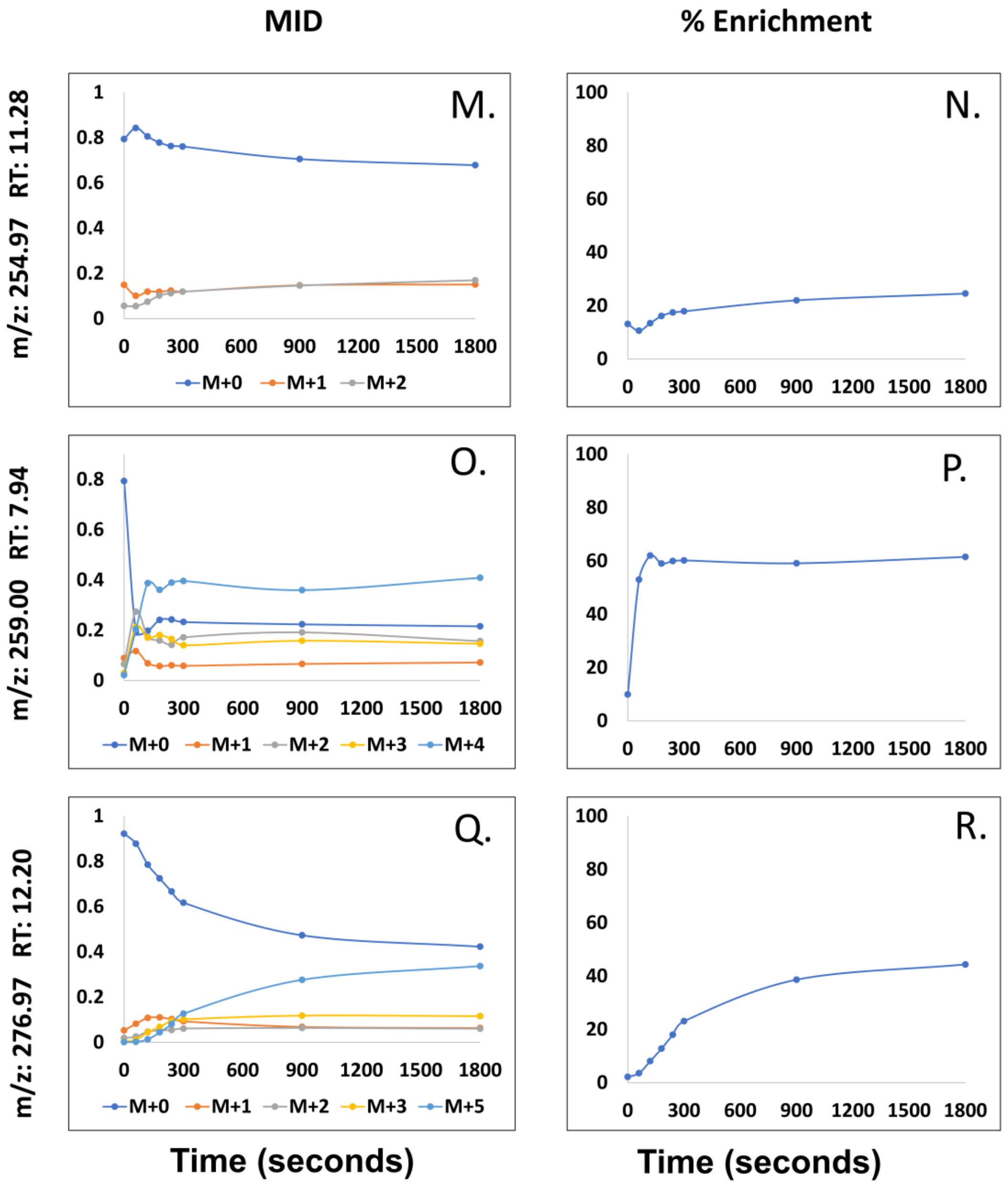

Figure S1 (contd.)

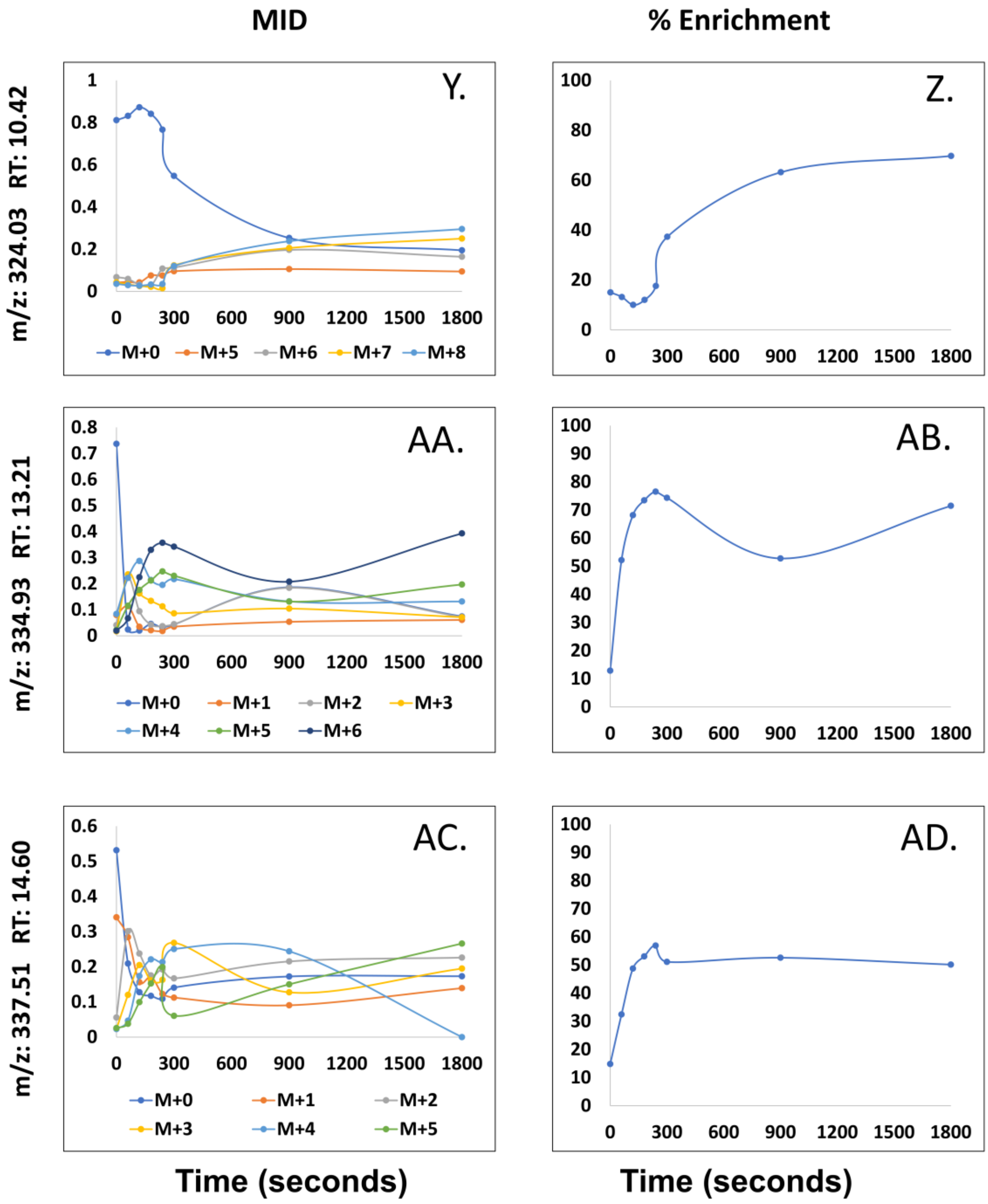

Figure S1 (contd.)

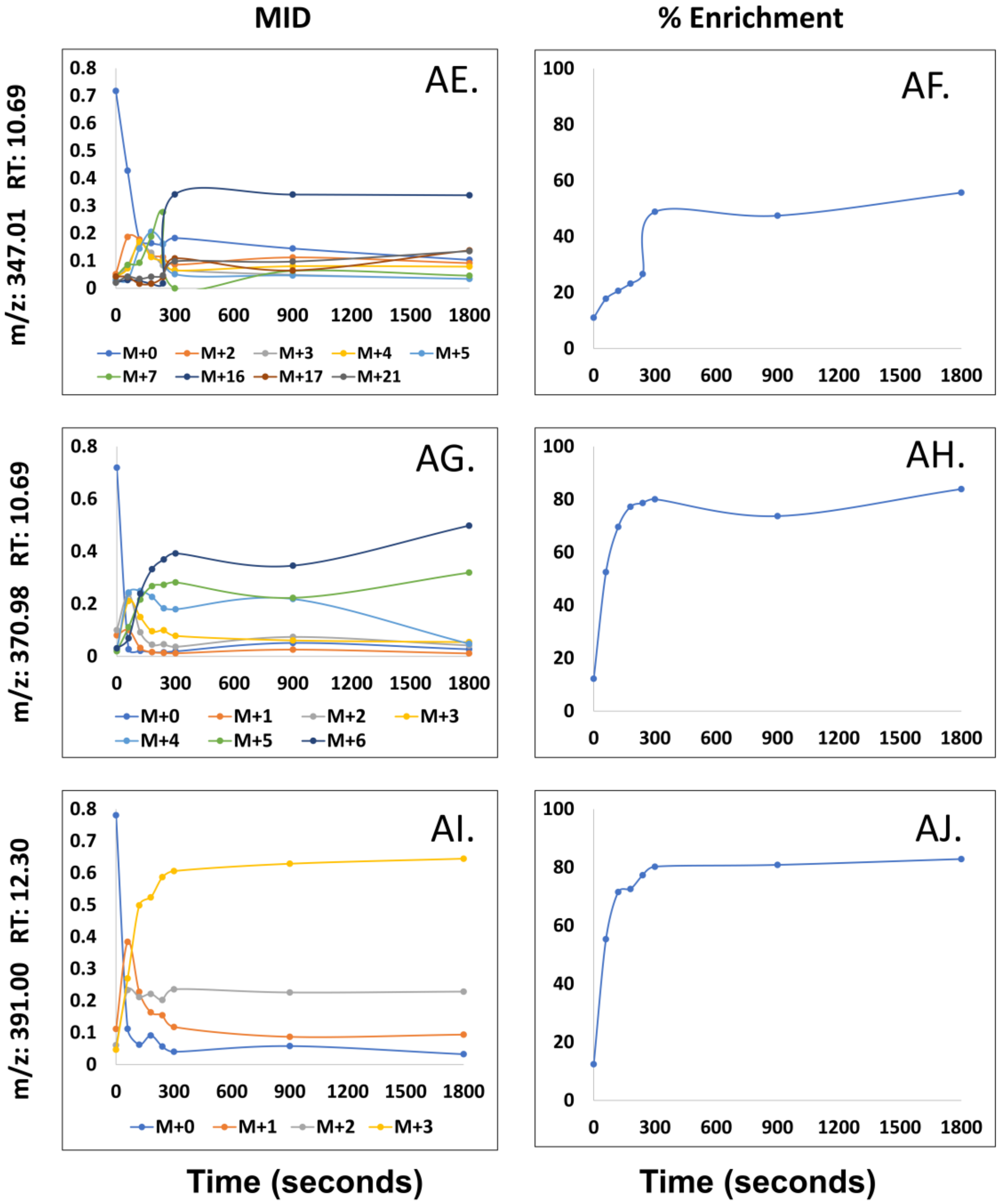

Figure S1 (contd.)

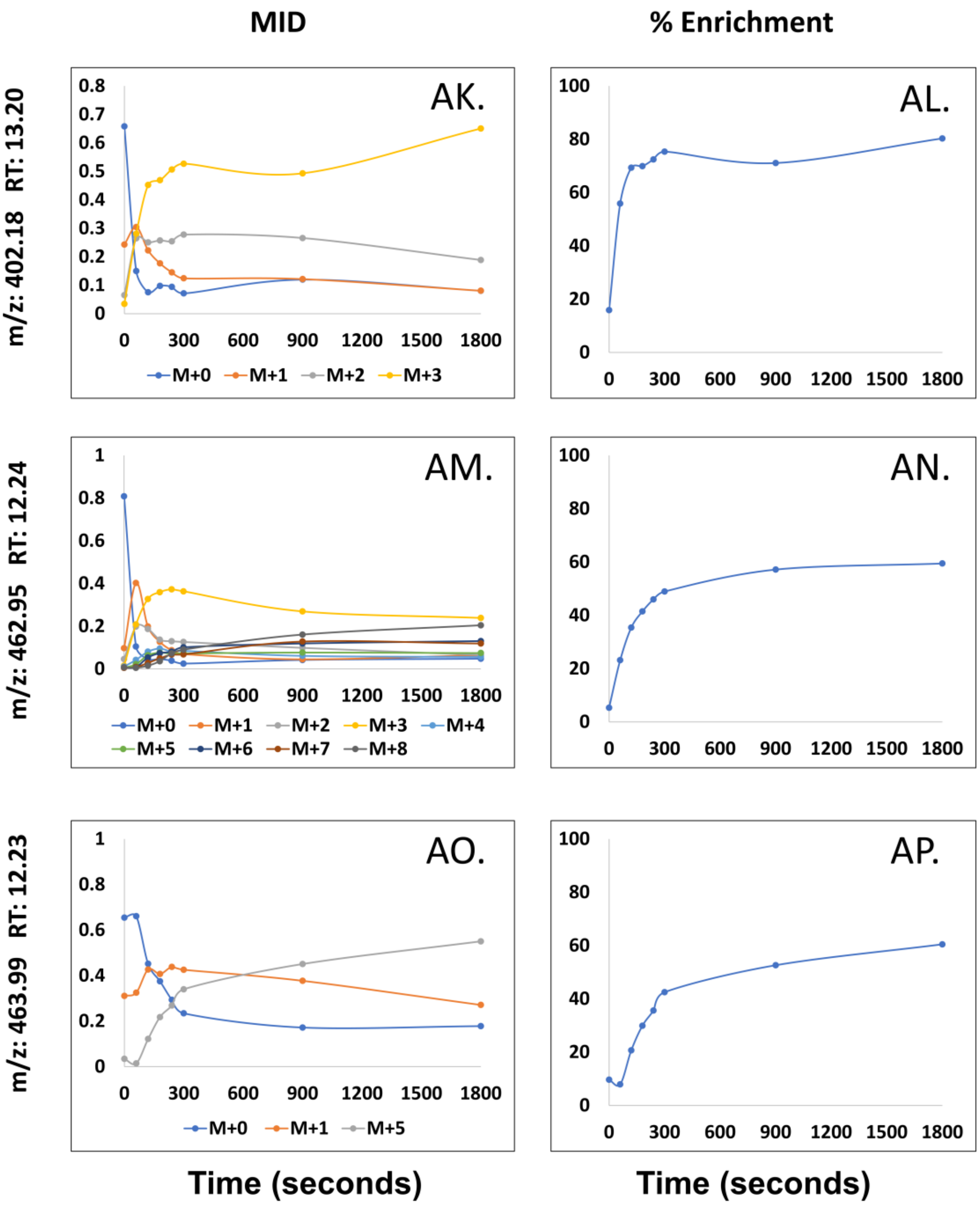

Figure S1 (contd.)

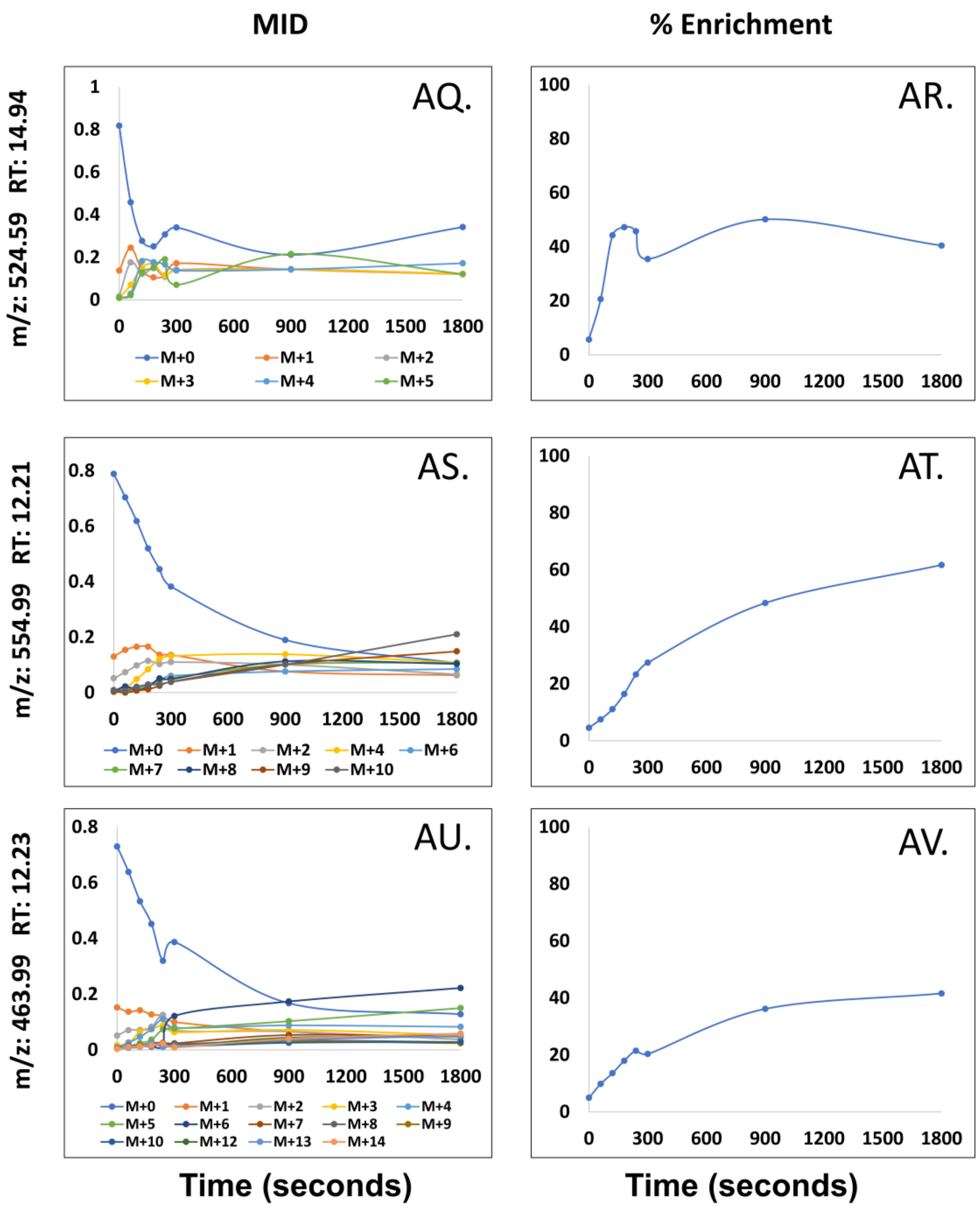

Figure S1 (contd.)

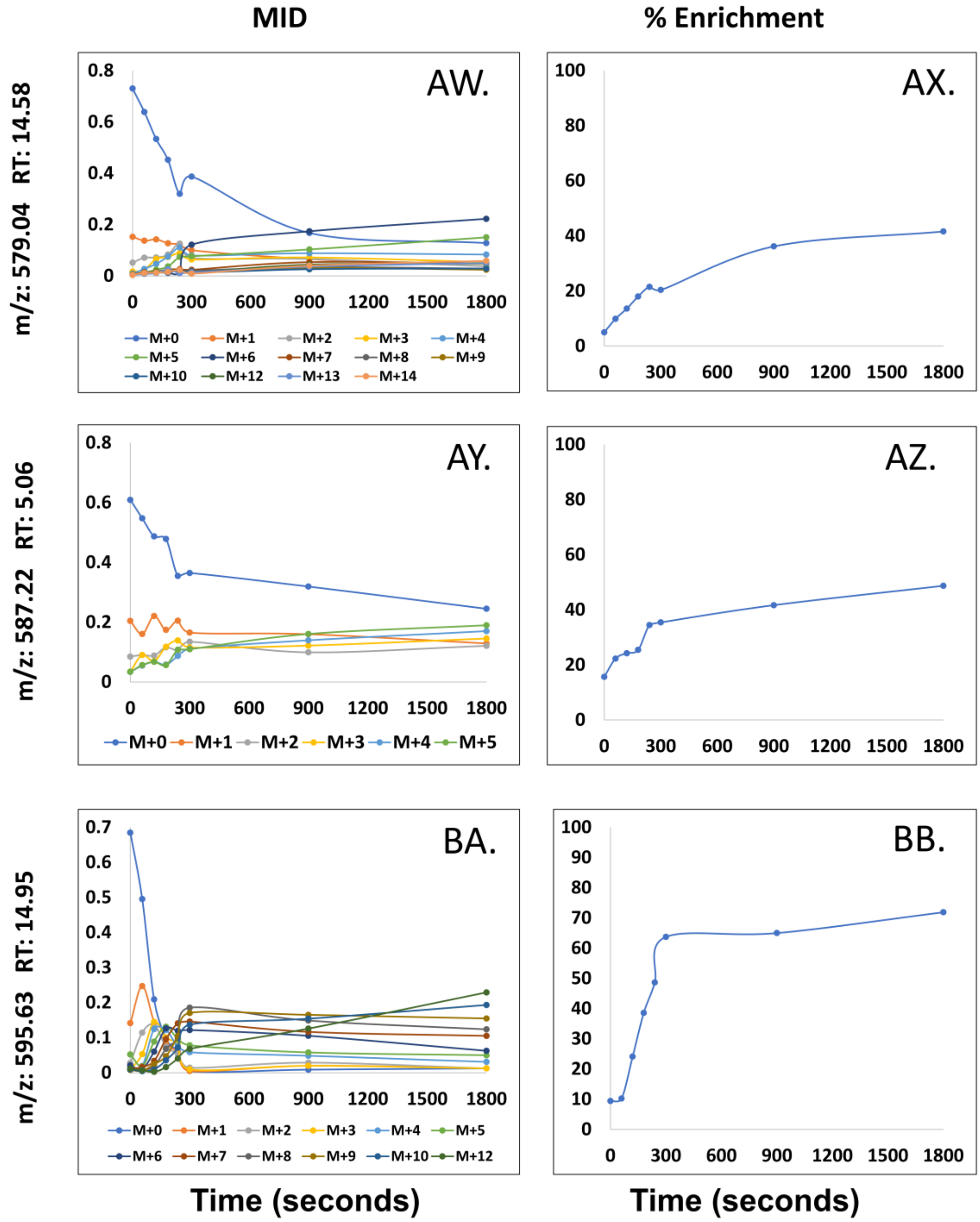

Figure S1 (contd.)

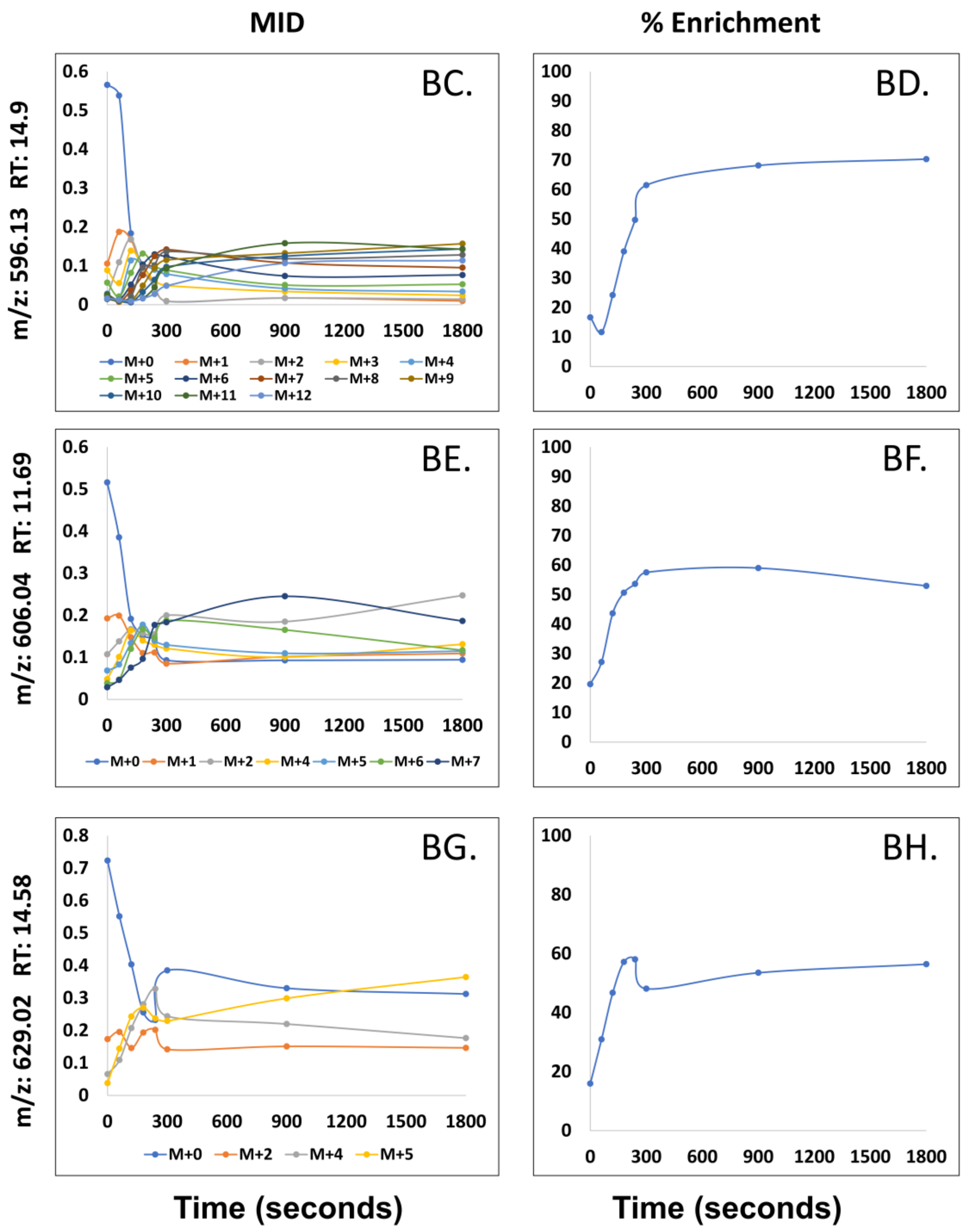

Figure S1 (contd.)

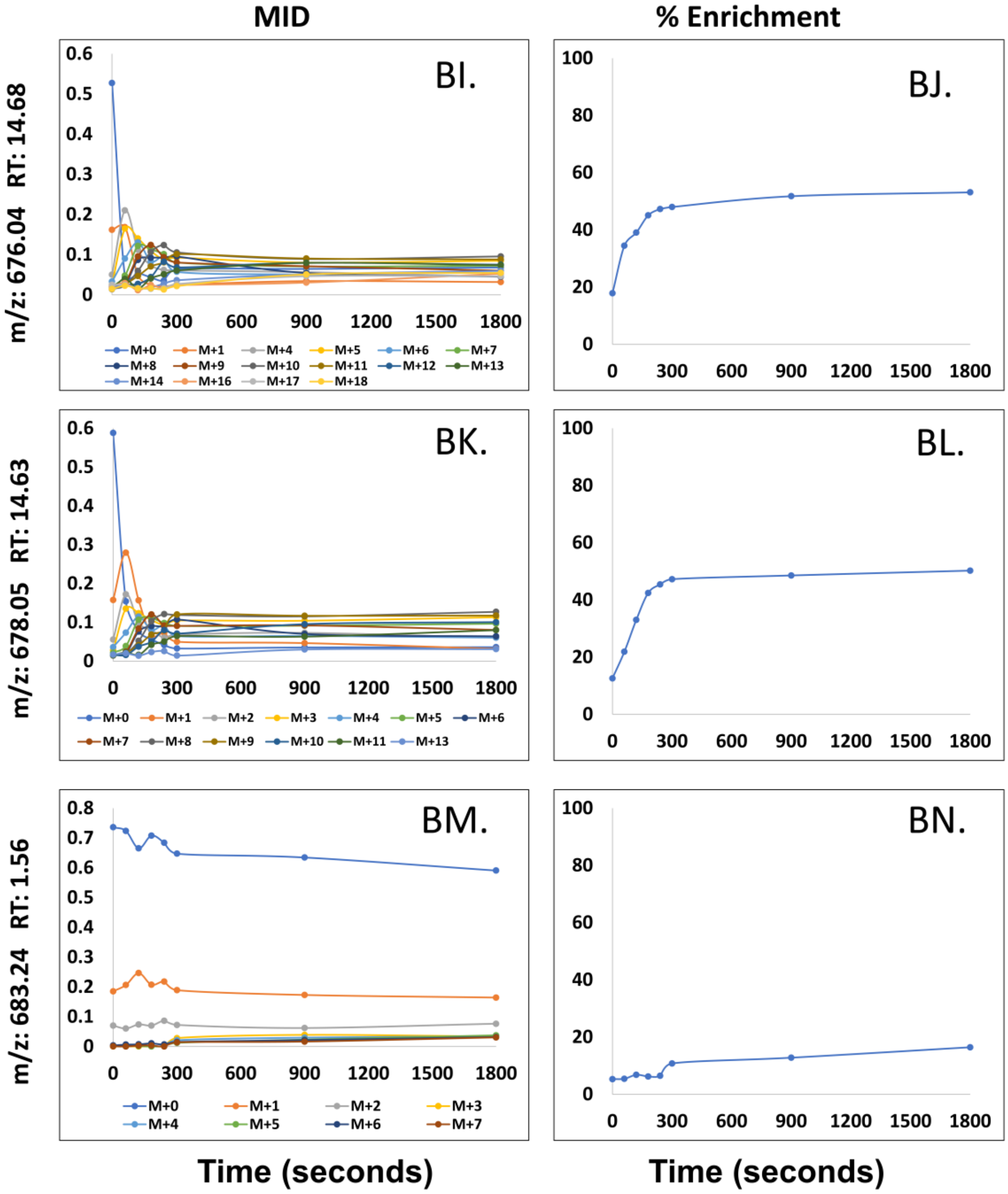

Figure S1 (contd.)

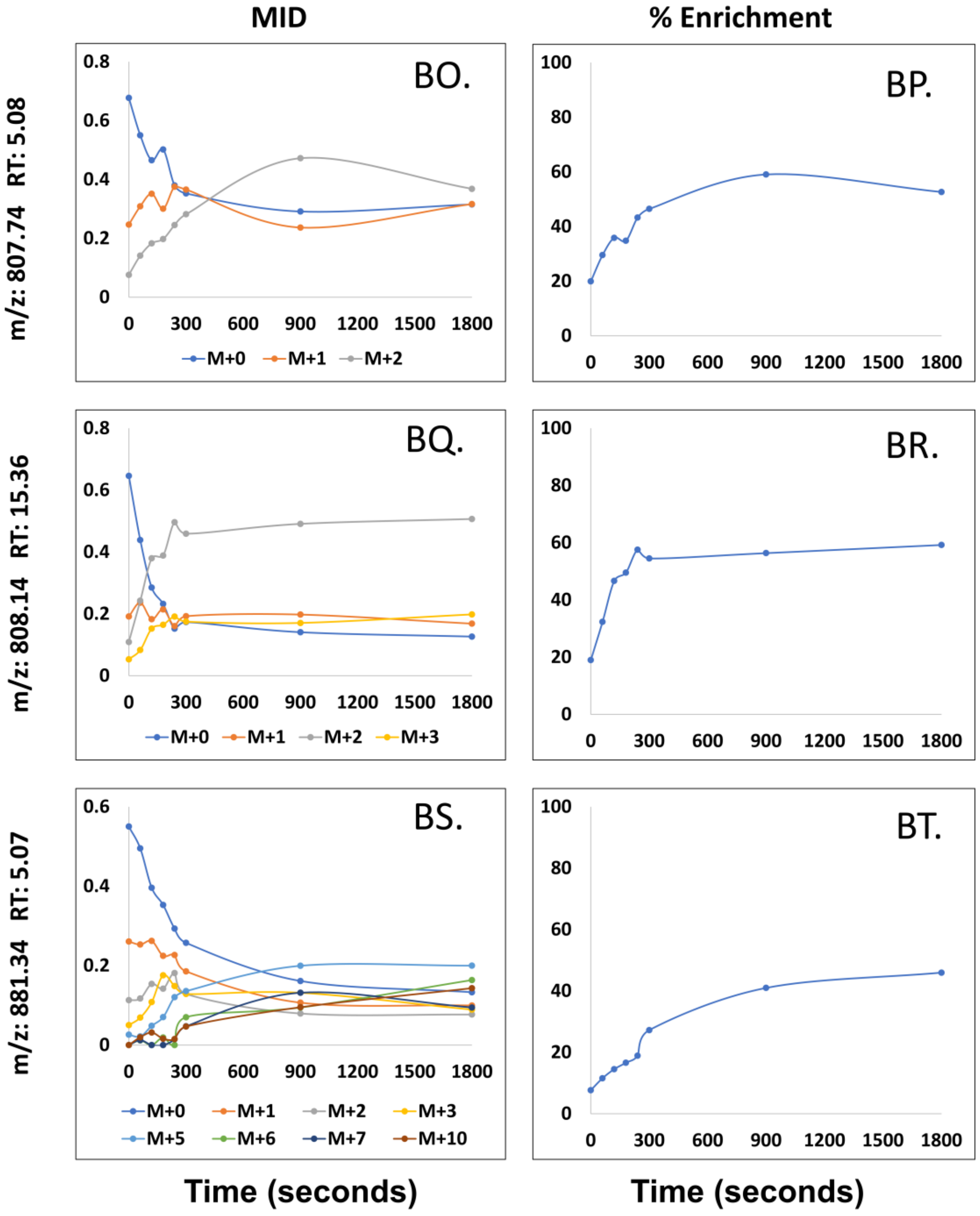

Figure S1 (contd.)

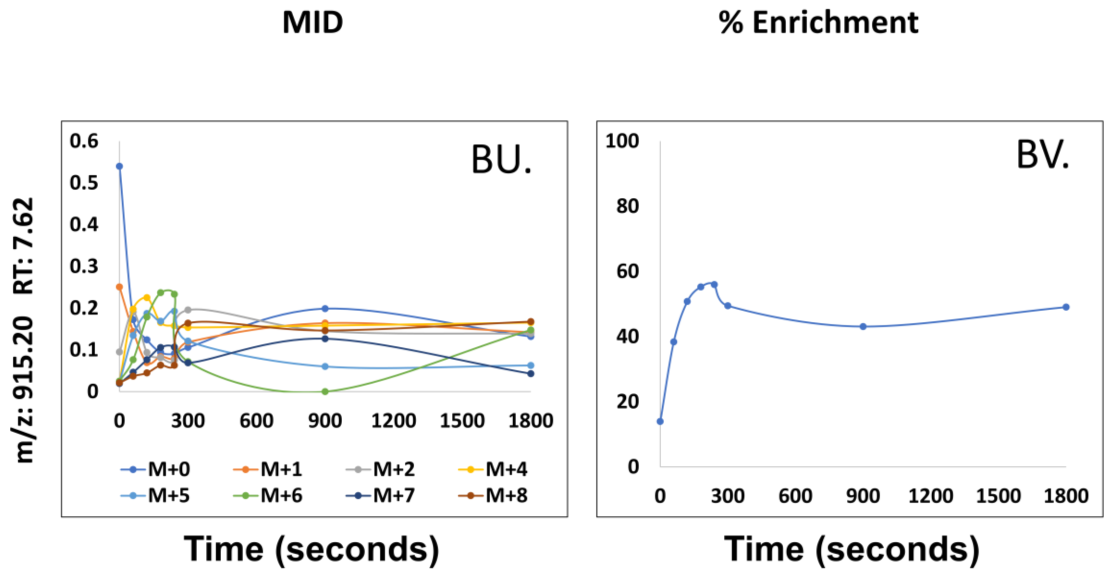

Figure S1 (contd.)

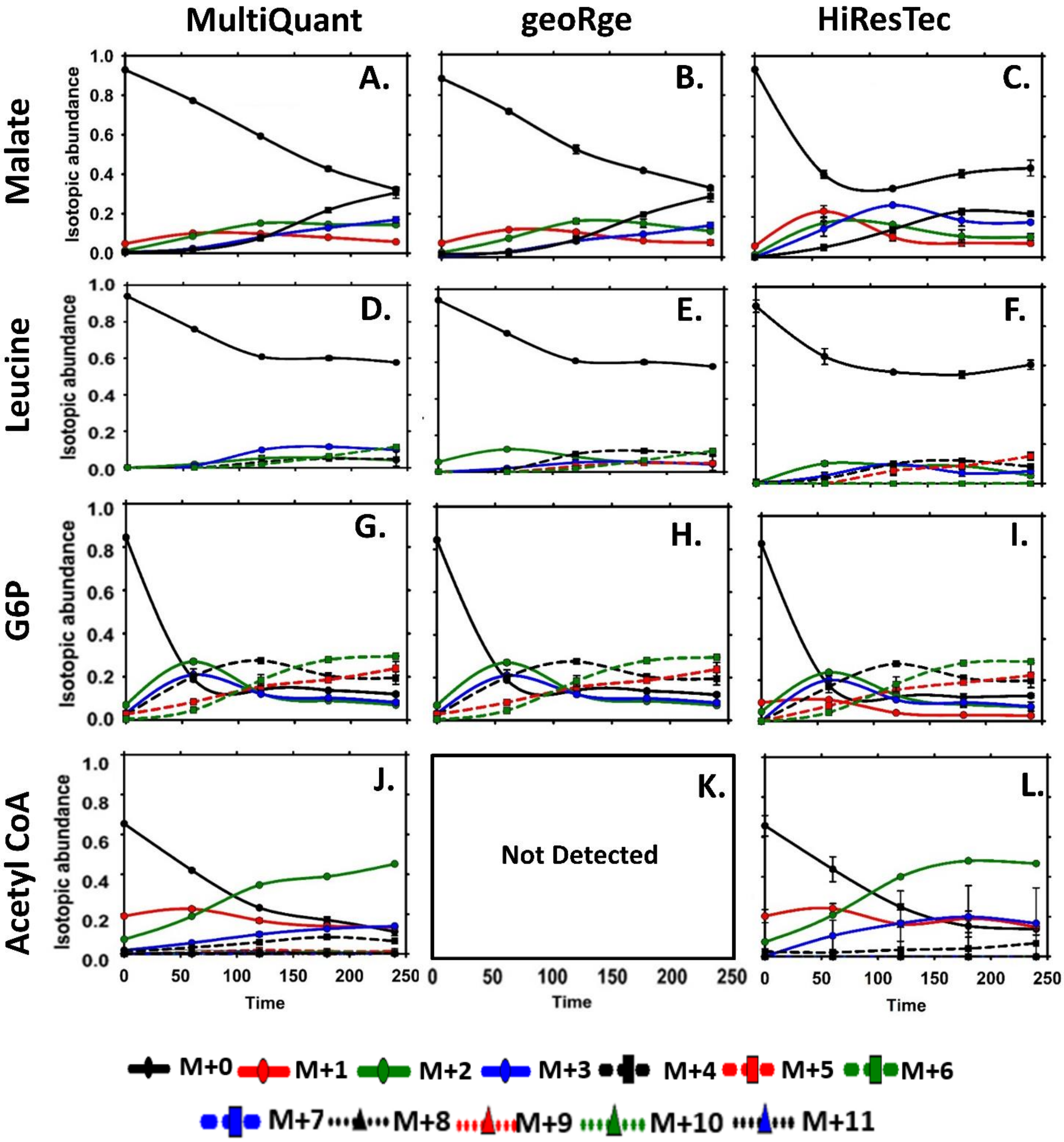

Figure S2: MID profiles of benchmarked metabolites, compared between reference (MultiQuant) and test software (geoRge and HiResTEC) for the *Synechococcus* sp. dataset.

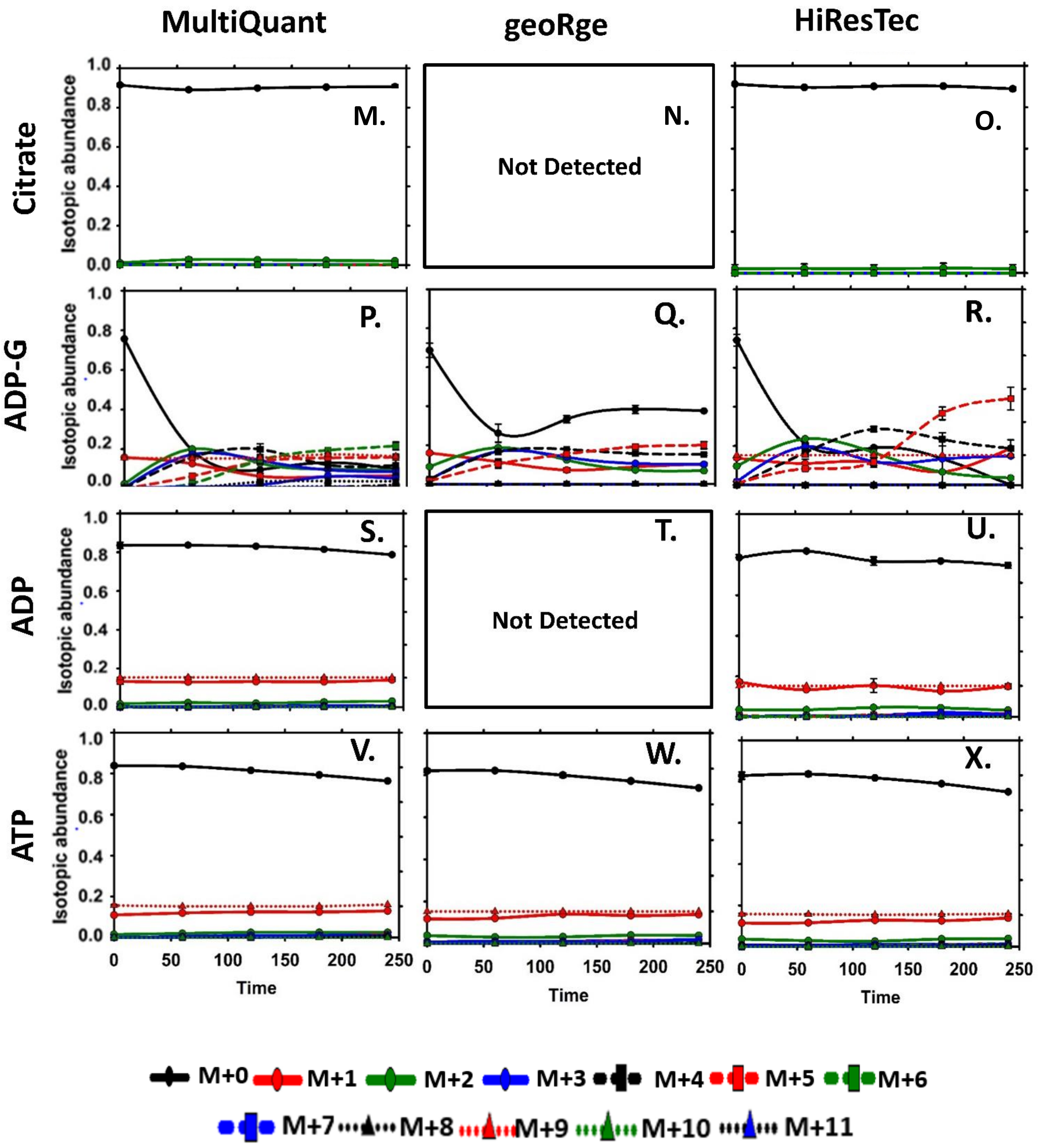

Figure S2 (contd.)

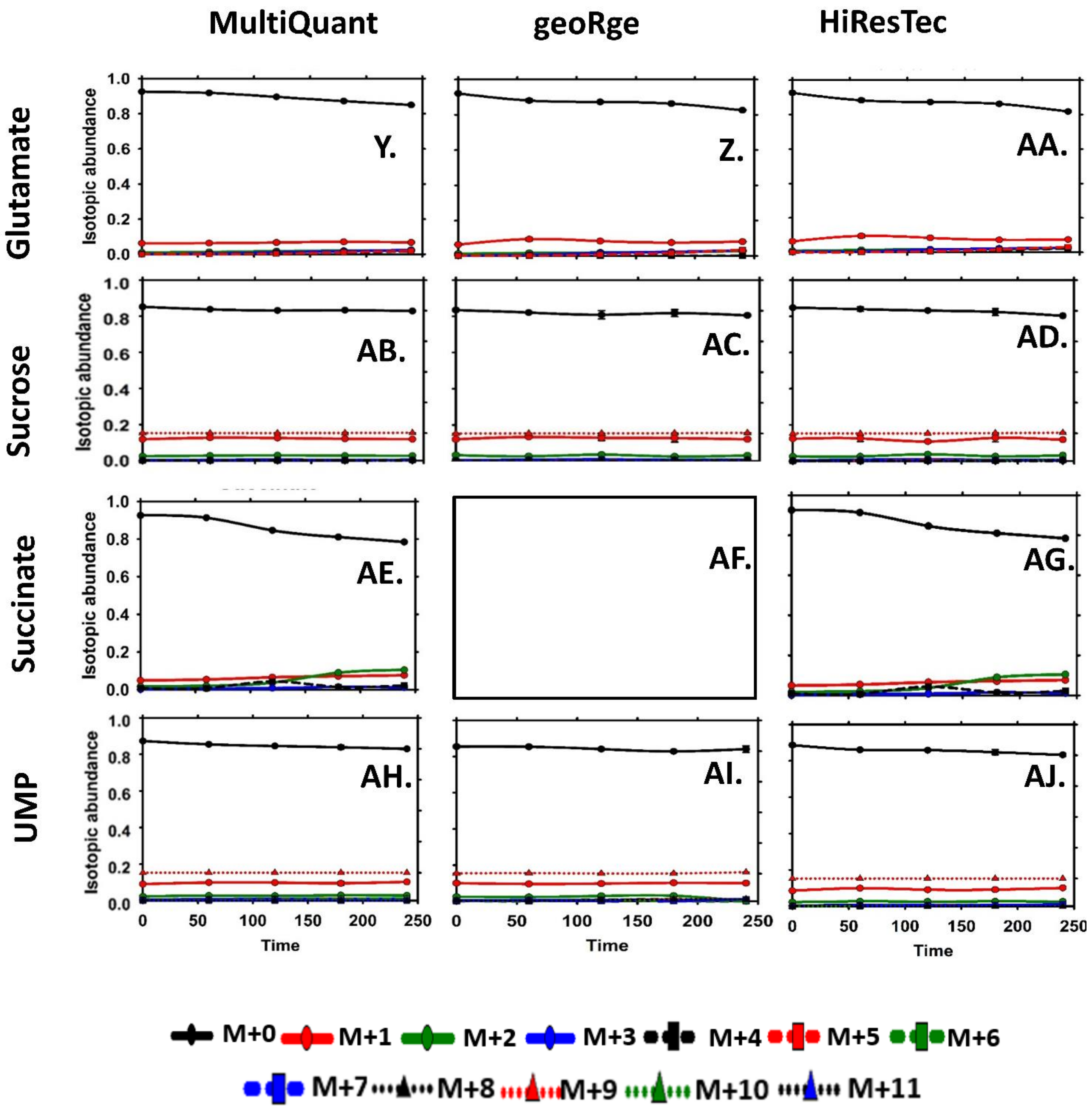

Figure S2 (contd.)

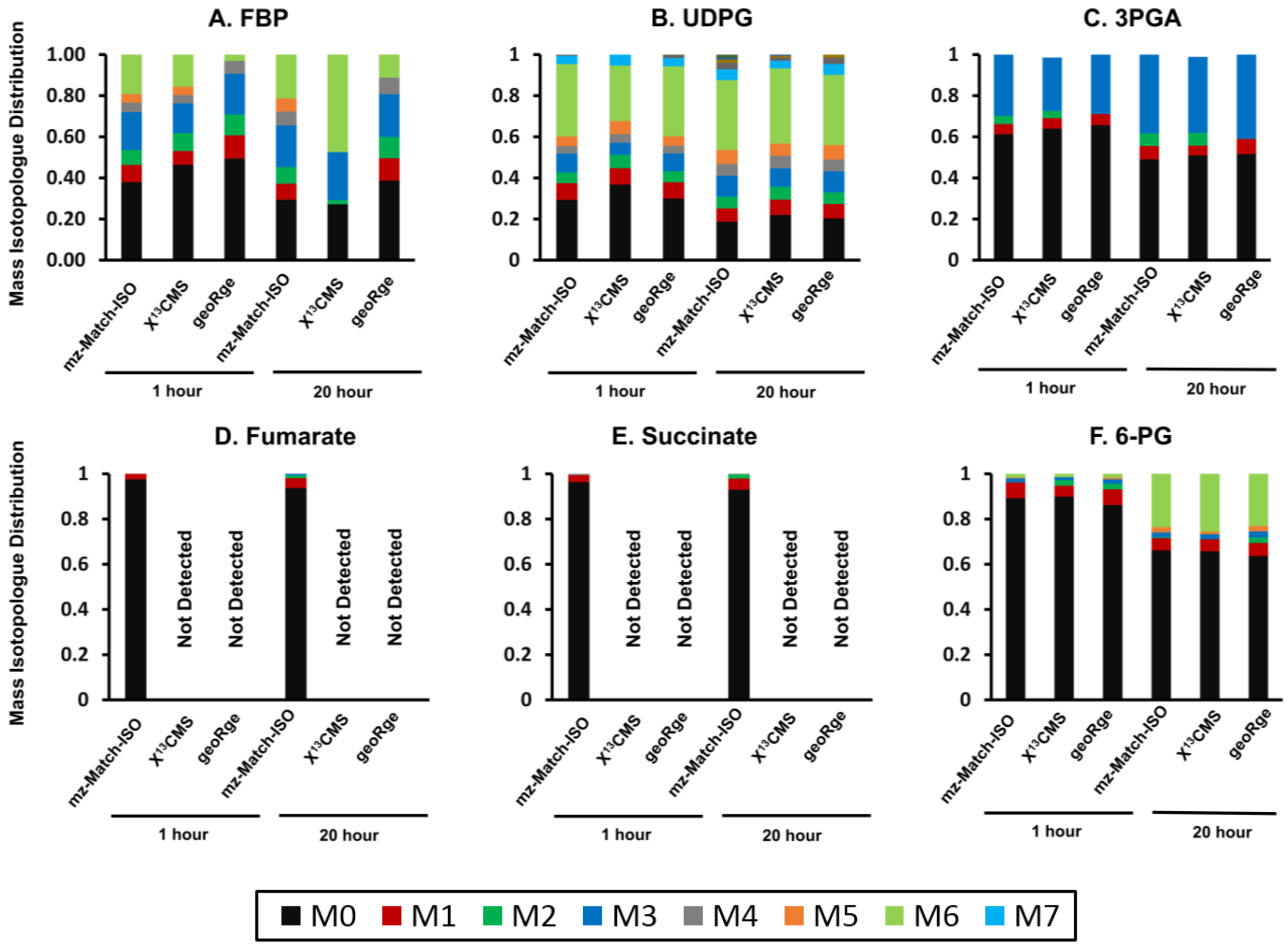

Figure S3: Comparison of MID profiles generated using the reference (mzMatch-ISO) and test software (geoRge and X[13]CMS) for the Reticulocytes dataset. Data collected at two time points, (1 and 20 hours) was considered.

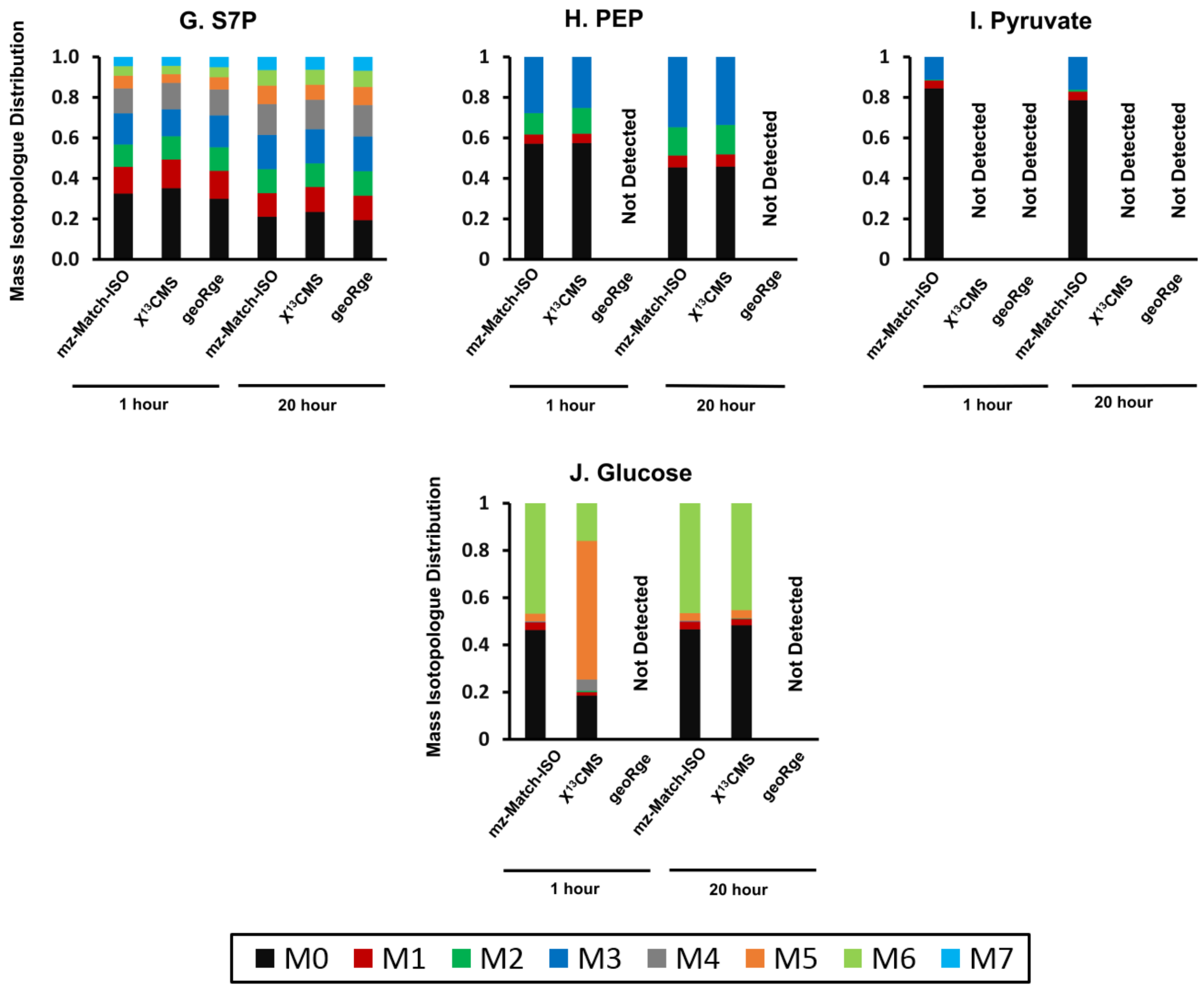

Figure S3 (contd.)

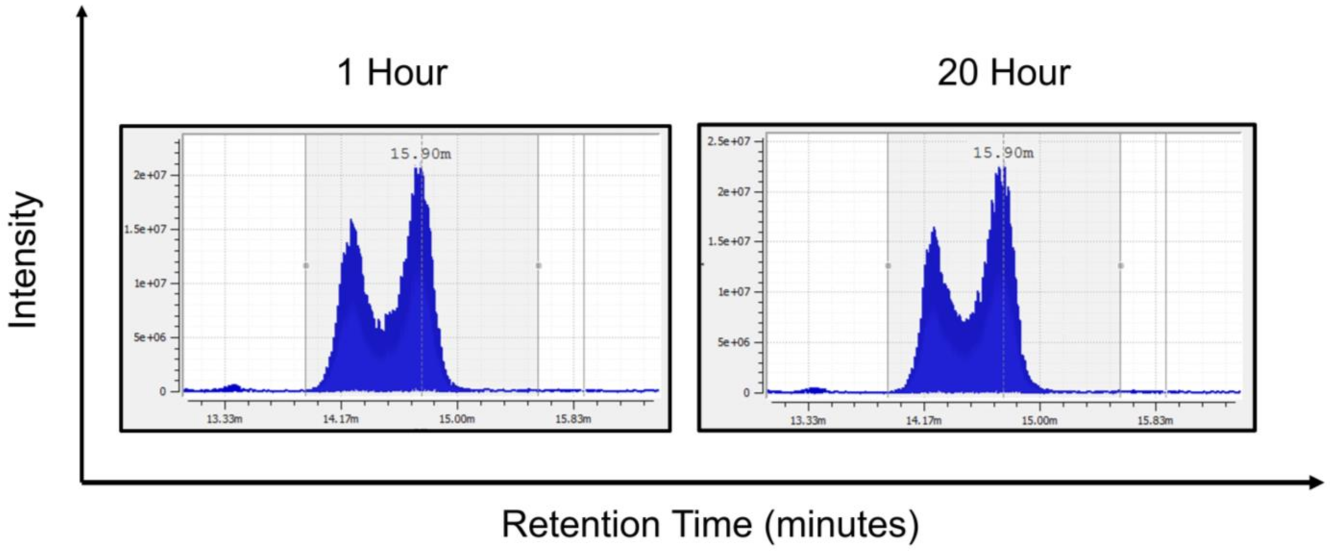

Figure S4: Extracted Ion Chromatogram (XIC) of Glucose for Reticulocytes Dataset for two time points 1 and 20 hour.

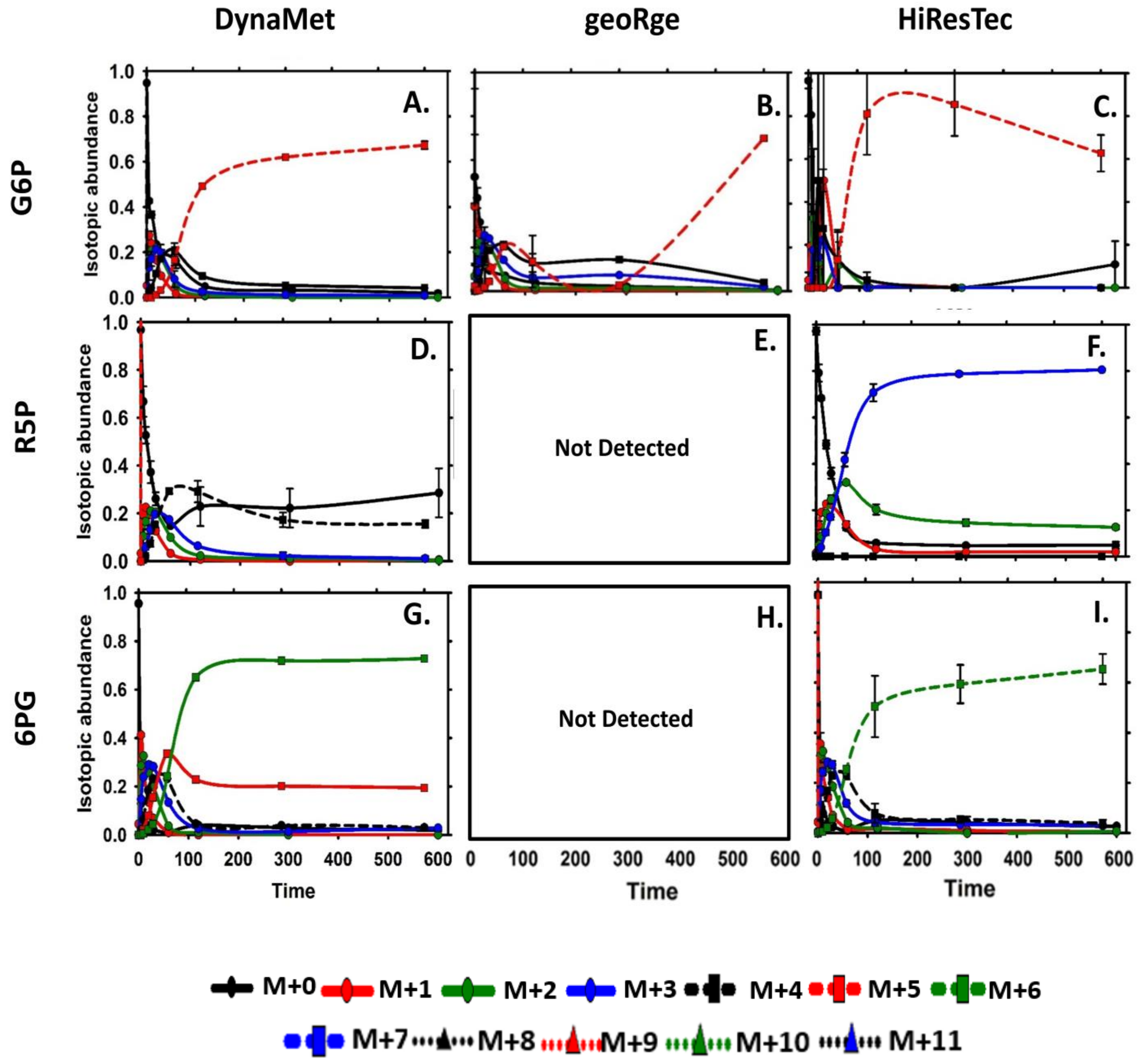

Figure S5: Comparison of MID profiles generated using the reference (DynaMet) and test software (geoRge and HiResTEC) for the Methanolicus dataset.

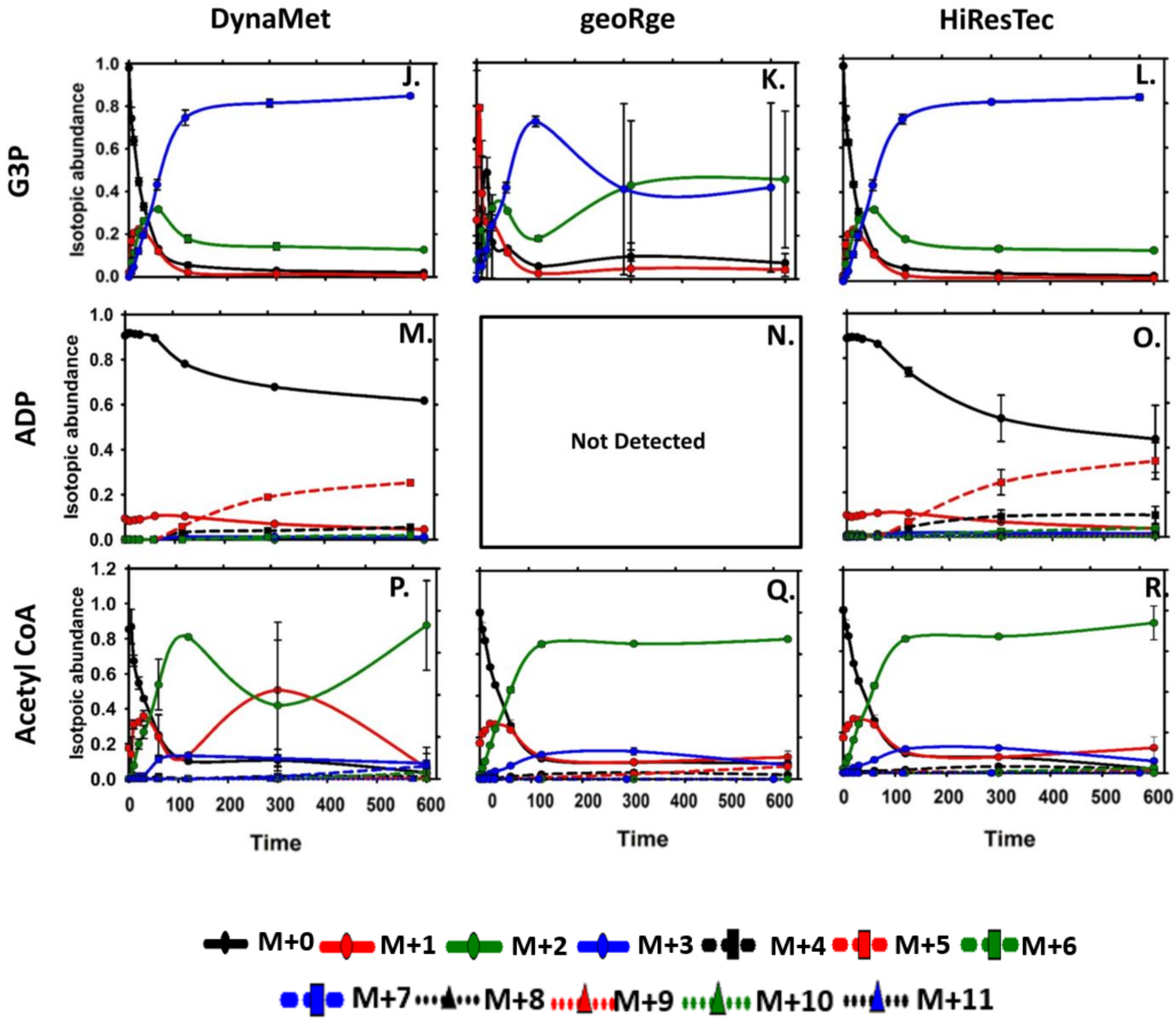

Figure S5 (contd.)

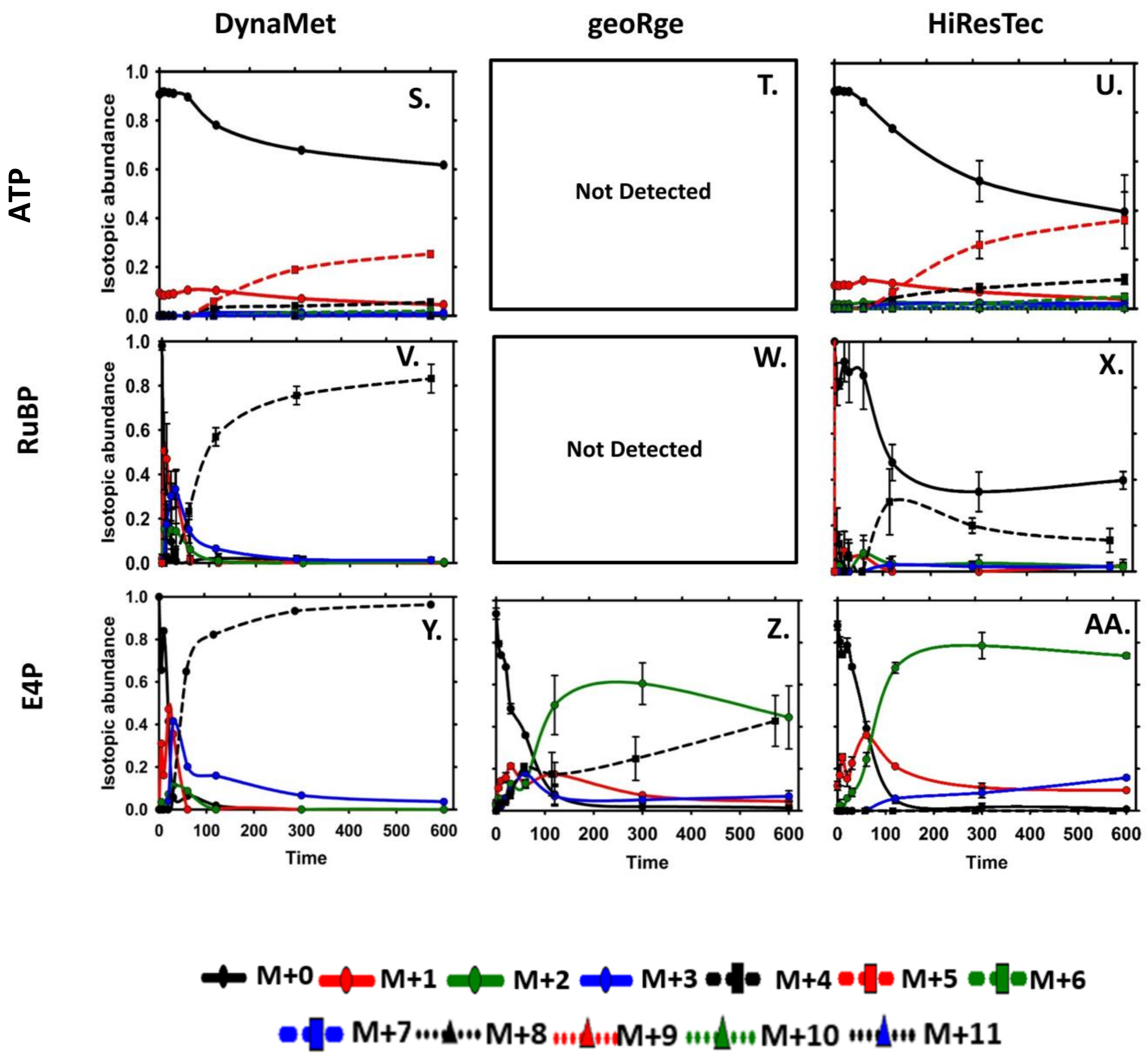

Figure S5 (contd.)

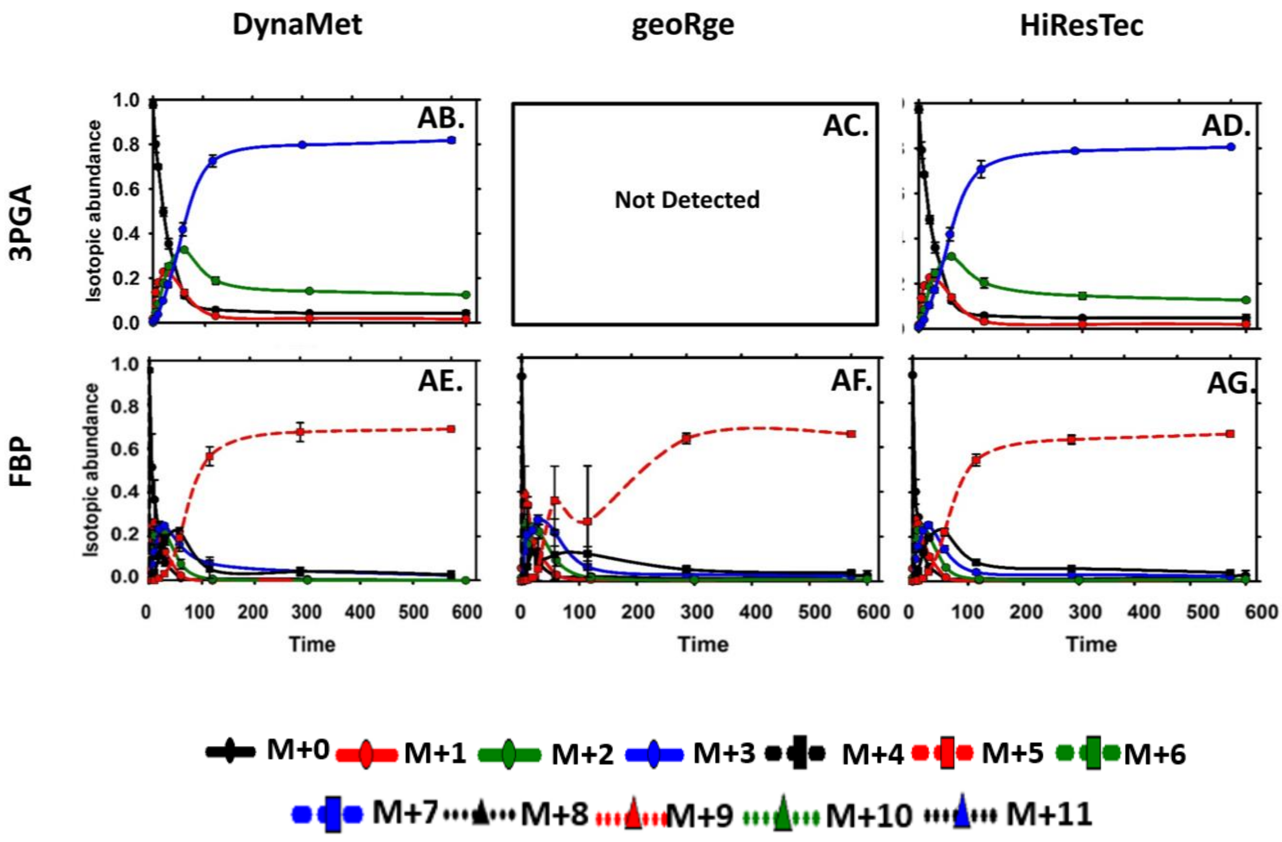

Figure S5 (contd.)
